# Genetic origins and diversity of bushpigs from Madagascar (*Potamochoerus larvatus*, family Suidae)

**DOI:** 10.1038/s41598-020-77279-5

**Published:** 2020-11-26

**Authors:** Carol Lee, Jenna Day, Steven M. Goodman, Miguel Pedrono, Guillaume Besnard, Laurent Frantz, Peter J. Taylor, Michael J. Herrera, Jaime Gongora

**Affiliations:** 1 Sydney School of Veterinary Science, Faculty of Science, The University of Sydney, Sydney, NSW 2006 Australia; 2 Field Museum of Natural History, Chicago, IL 60605 USA; 3 Association Vahatra, 101 Antananarivo, Madagascar; 4 UMR ASTRE, INRAE, CIRAD, Université de Montpellier, 34398 Montpellier Cedex 5, France; 5 CNRS, UPS, IRD, Laboratoire Evolution et Diversité Biologique, UMR5174, Université Toulouse III Paul Sabatier, 31062 Toulouse, France; 6 Palaeogenomics Group, Department of Veterinary Sciences, Ludwig Maximilian University, Munich, Germany; 7 School of Biological and Chemical Sciences, Queen Mary University of London, London, UK; 8 School of Mathematical and Natural Sciences, University of Venda, Thohoyandou, Limpopo Province South Africa; 9 Afromontane Research Unit and Zoology Department, University of the Free State, Qwa Qwa campus, Phuthaditjhaba, 9866 South Africa; 10 Archaeological Studies Program, University of the Philippines Diliman, 1101 Quezon City, Philippines

**Keywords:** Adaptive immunity, Taxonomy, Ecological genetics, Evolutionary ecology, Molecular ecology

## Abstract

The island of Madagascar, situated off the southeast coast of Africa, shows the first evidence of human presence ~ 10,000 years ago; however, other archaeological data indicates a settlement of the modern peoples of the island distinctly more recent, perhaps > 1500 years ago. Bushpigs of the genus *Potamochoerus* (family Suidae), are today widely distributed in Madagascar and presumed to have been introduced from Africa at some stage by human immigrants to the island. However, disparities about their origins in Madagascar have been presented in the literature, including the possibility of endemic subspecies, and few empirical data are available. Furthermore, the separation of bushpigs in Madagascar from their mainland relatives may have favoured the evolution of a different repertoire of immune genes first due to a founder effect and then as a response to distinct pathogens compared to their ancestors. Molecular analysis confirmed the species status of the bushpig in Madagascar as *P. larvatus*, likely introduced from the central region of southern Africa, with no genetic evidence for the recognition of eastern and western subspecies as suggested from previous cranial morphology examination. Investigation of the immunologically important *SLA-DQB1* peptide-binding region showed a different immune repertoire of bushpigs in Madagascar compared to those on the African mainland, with seventeen exon-2 haplotypes unique to bushpigs in Madagascar (2/28 haplotypes shared). This suggests that the MHC diversity of the Madagascar populations may have enabled Malagasy bushpigs to adapt to new environments.

## Introduction

The first evidence of human presence in Madagascar, situated off the south-eastern coast of Africa, dates from close to 10,000 years ago^1^. Subsequently, the island was settled by Austronesians approximately 1500–3000 years ago and then soon thereafter by Bantu groups from East Africa, but evidence of this remains unclear^2–4^. The bushpig (*Potamochoerus larvatus*; Suidae, Artiodactyla) is suggested to have been introduced to Madagascar from eastern Africa by early sea navigators who settled on the island^5,6^. The earliest archaeological evidence for the bushpig on Madagascar dates to the tenth to thirteenth centuries^7^. On other regional islands, archaeological records show evidence of bushpigs on the Comoro Islands from the ninth to tenth centuries^8,9^. However, there are large gaps in the paleontological record of Madagascar between the Late Cretaceous and the Late Pleistocene (~ 66 million to 120,000 years ago)^10^, as well as the Holocene (the earliest possible *Potamochoerus* introduction to Madagascar), hampering the determination of when and where the species was first established on the island and whether this predates human arrival. Genetic evidence is useful to assess where the *Potamochoerus* lineage(s) on the island originated from and determine whether their separation resulted in any significant genetic differentiation from their mainland counterparts, in particular, genomic regions that underlie the adaptive immune response to diseases and other environmental challenges^11,12^. Bushpigs are classified as Least Concerned on the IUCN Red List^13^ and a pest as they damage farmlands and prey on endemic Malagasy species^14^. However, they are an important meat source and income for some rural populations in Madagascar, and seed dispersers of some native flora^14^. Understanding their genetic diversity is essential for sustainable population management, protecting local agriculture, and the health of the local ecosystem.

**Figure 1:**
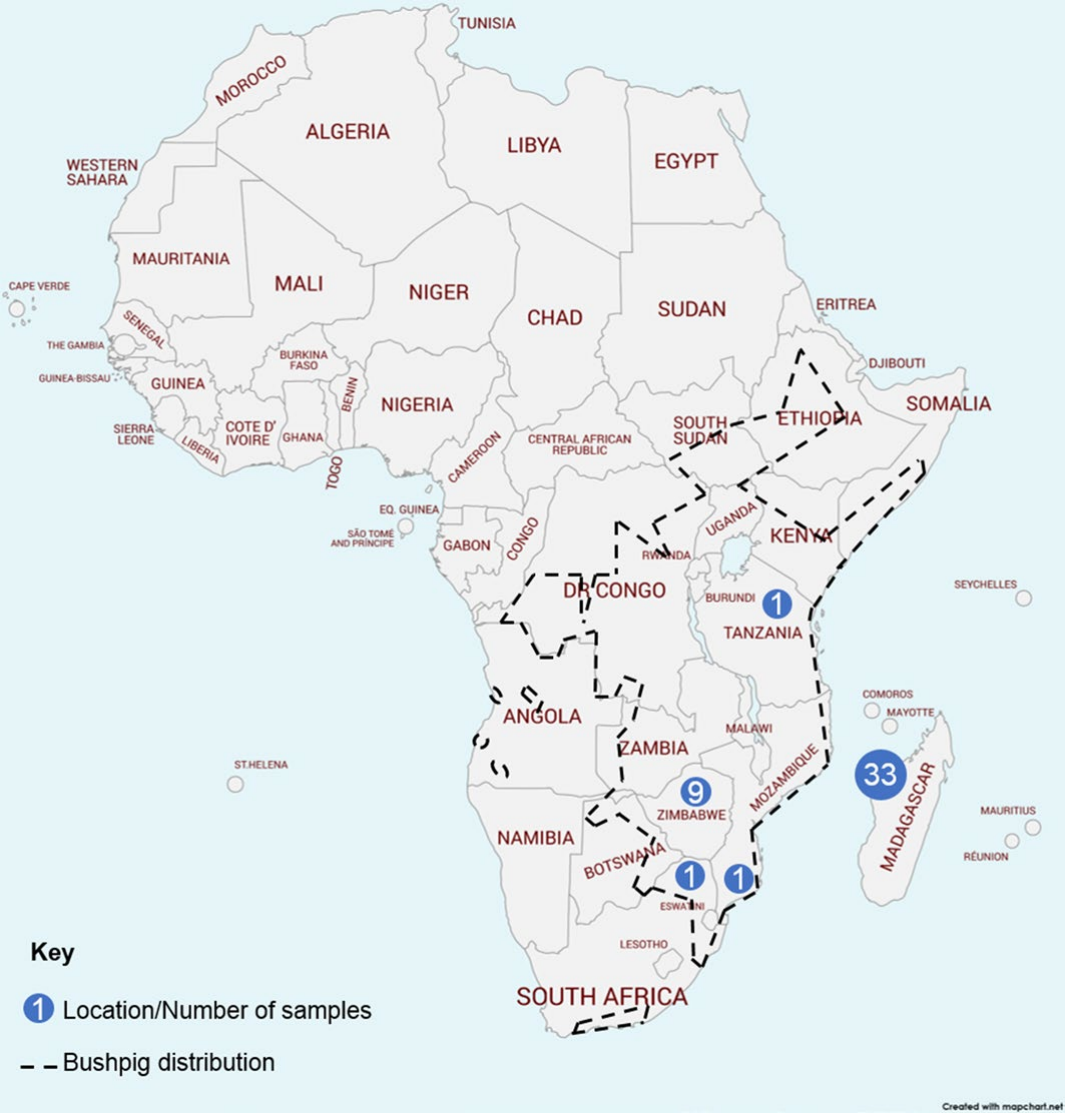
Map of native bushpig distribution as illustrated in the IUCN. Map is a modified version by mapchart.net.

The genus *Potamochoerus* includes two separate species: *P. larvatus* distributed in portions of eastern, central, and southern Africa (Fig. 1) and *P. porcus* occurring in the Congo Basin and western Africa. Based on few specimens and mostly cranio-dental characteristics, early taxonomists proposed that the western and eastern populations of bushpigs in Madagascar should be separated into two endemic subspecies, *P. l. larvatus* from the West and *P. l. hova* from the East^15^. However, other taxonomists have concluded that bushpigs from Madagascar are morphologically indistinguishable from African specimens of *P. larvatus*^16^, in which provisional subspecies separating bushpigs from eastern Africa and those from southern African and Madagascar, are distinguished based on cranial differences and body colour^15^. Due to the considerable phenotypic variation in mainland African *Potamochoerus*, both within and between populations, the origins of animals in Madagascar have been difficult to discern based on cranio-dental characters. Although it has been proposed that the eastern bushpigs of Madagascar may have originated from southern African populations based on similarities to *P. l. koiropotamus* (ranging from Tanzania, to northern South Africa)^15,17^, western bushpigs of Madagascar are apparently more similar to those found on the Comoro Islands, which are also presumably introduced, and appear larger than their eastern and mainland equivalents^15,18,19^. For Madagascar, it is possible that bushpigs were introduced to the island prior to the ninth century AD and the gradual spread of Islam in East Africa may have increased the translocation of bushpigs to offshore islands by hunter-gathers of other communities^8,20^. This could have occurred via the Comoros, perhaps using the Southern Equatorial which connects the southeast trade winds, as (limited) evidence of *Potamochoerus* has been found at M’Bachile on Grande Comore dating to the ninth–tenth century^9^. Although the North Mozambique current may have been used, this route is more treacherous for sailing vessels due to the reefs and sandbars along this current^21^.

Wild suids in Africa—*Phacochoerus* and *Potamochoerus* spp.—have important ecological implications as hosts or reservoirs in a range of diseases such as African swine fever virus (ASFV)^22–25^. ASFV can devastate domestic pig populations and cause huge economic loss for farmers^26,27^. Bushpigs can be asymptomatically infected by ASFV but previous studies have indicated that bushpigs in Madagascar have an insignificant role in ASFV transmission due to the absence of circulating ASFV, anti-ASFV antibodies, nor antibodies to tick vectors (*Ornithodoros* spp.)^11^. The separation of Malagasy bushpigs from the mainland has raised questions about the change in their susceptibility to ASFV due to genetic drift and an absence of natural infection. In general, the introduction of few founders on islands often have adverse effects on fitness of subsequently established populations^12,28^. This is of particular interest as the relevant antibodies were detected in domestic pigs from shared habitats and occasional contacts between these two groups and arthropods in the environment could play a role in maintaining ASFV in Madagascar^11,23^. The major histocompatibility complex (MHC) is an important part of adaptive and innate immunity in vertebrates^29^ and has been studied comprehensively in model species including the domestic pig (*Sus scrofa*) and humans^30–33^. Genetic diversity in this genomic region contributes to individual and population fitness in natural populations through pathogen recognition^12,34^. In particular, the antigen-binding sites (ABS) within the class I and class II MHC molecules are responsible for self and non-self recognition and present peptides to the CD4+ T lymphocytes for surveillance^35,36^. The high genetic diversity in the MHC can be caused by different evolutionary mechanisms such as gene duplication and pathogen-mediated co-evolution and consequently diversification of the ABS through balancing and positive selection for advantageous alleles^37–39^. Balancing selection has been described as one of the major factors driving this diversity as a higher number of different alleles (heterozygote advantage) could bind and present different antigens^40,41^. Thus, examining the MHC of bushpigs from Madagascar could provide an opportunity to better understand how these mechanisms have changed the MHC repertoire of *Potamochoerus* over space and time.

In this study, the origins and taxonomic position of bushpigs from Madagascar were assessed using the Sanger sequencing data of mitochondrial (control region; CR, and *cytochrome b; cytb*) and nuclear markers (glucosephosphate isomerase-processed pseudogene; *GPIP*, and melanocortin 1 receptor; *MC1R*). A range of suids and peccaries (nine species), and specimens of *Potamochoerus* from Madagascar were sequenced in this study. Due to the varied bioclimatic regime found in different areas of the island^42^, as compared to mainland Africa, we sequenced three MHC loci to assess whether the separation of Malagasy bushpigs generated a different repertoire of immune genes compared to mainland bushpigs. These genetic analyses provide insights into the diversity of the Malagasy bushpig adaptive immune system in comparison to mainland bushpigs since their separation. Similar comparisons could be used to assess the fitness and historical changes incurred by other species translocated to Madagascar.

## Materials and methods

### Sampling

Genomic DNA was extracted from blood and tissue samples from a total of 80 individuals (Table 1). Samples included 55 bushpigs (*P. larvatus*) consisting of 13 specimens from mainland Africa (Zimbabwe, Tanzania-museum specimen, South Africa; SA), and 42 specimens from regions of “West” Madagascar (general Mahajanga Province area, which includes a museum specimen collected near the Ambohijanahary Special Reserve and two other protected areas: the Menabe Antimena and the Ankarafantsika National Park), “East” Madagascar (Andasibe-Mantadia protected area), and “North” Madagascar (museum specimen collected from Forêt d*'*Antsahabe, Antsiranana Province) (Fig. 1). Genomic DNA for non-museum specimens and museum specimens were extracted using the DNeasy Blood & Tissue Kit (Qiagen, France) and phenol/chloroform method, respectively. Museum specimens were provided by the Field Museum of Natural History, Illinois, USA. Samples were collected under permission from the Direction Générale des Eaux et Forêts de Madagascar (Supplementary Table S1). The tissue samples were from recently killed bushpigs found during field trips and skulls were kept as voucher specimens. Although Madagascar no longer uses the province system in an administrative manner, we maintain these names for geographical descriptive purposes. For phylogenetic comparison and to provide an overview on the similarity of immune genes, the study also included sampling of three red river hogs (*P. porcus*), 16 collared peccaries (*Pecari tajacu*), and one specimen of the following species: European wild boar (*S. scrofa*), Bornean beared pig (*S. barbatus*), Celebes warty pig (*S. celebensis*), Buru babirusa (*Babyrousa babyrussa*), common warthog (*Phacochoerus africanus*), and giant forest hog (*Hylochoerus meinertzhageni*) (Table 1). Outgroup species were obtained from previous studies^43–45^.

**Table 1:** Details of pig and peccary samples used in this study.

Species name	Common name	*N*	Distribution	Sampling location
*Potamochoerus larvatus*	Bushpig	18^a^	Sub-Saharan Africa (mainland)	Tervuren (Belgium), Duisburg Zoo (Germany), Soutpansberg (South Africa), Zimbabwe, KwaZulu-Natal (South Africa), Tanzania
	Bushpig	38^a^	Madagascar	“West” Madagascar
	Bushpig	4	Madagascar	“East” Madagascar
	Bushpig	1	Madagascar	“North” Madagascar
*Potamochoerus porcus*	Red river hog	3	Sub-Saharan Africa	Tervuren (Belgium), Rotterdam Zoo (Netherlands), Duisburg Zoo (Germany)
*Phacochoerus africanus*	Common warthog	1	Sub-Saharan Africa	Iwaba (Zimbabwe)
*Hylochoerus meinertzhageni*	Forest hog	1	Sub-Saharan Africa	Uganda
*Sus scrofa*	Wild boar	1	Eurasia	Yorkshire Farm (UK)
*Sus barbatus*	Bornean bearded pig	1	Southeast Asia	ZSL Animal Hospital (UK)
*Sus celebensis*	Sulawesi warty pig	1	Southeast Asia	Sulawesi mainland (Indonesia)
*Babyrousa babyrussa*	Babirusa	1	Southeast Asia	Edinburgh Zoo (UK)
*Pecari tajacu*	Collared peccary	20^a^	South America	Macagual and Barranquilla Zoo (Colombia)

The species common name, natural distribution, sampling location and number of samples (*N*) are indicated.

^a^*P. larvatus* samples included replicates for six individuals and *P. tajacu* included replicates for three individuals (one individual replicated twice).

### Selection of target regions

To examine the phylogenetic position of bushpigs from Madagascar, the mitochondrial CR^46^ and *cytb*^47^, and the nuclear *GPIP* region^48,49^ and *MC1R*^50^ region were sequenced. Mitochondrial markers have been extensively used to infer evolutionary relationships of many organisms^51–54^. The control region and *cytochrome b* has been particularly valuable in Suidae and Tayassuidae; identifying differences amongst species and pig breeds^48,55–57^. The mitochondrial markers were concatenated as they are essentially a single, non-recombining locus. The nuclear marker *GPIP* was sequenced due to minimal selection pressures and previous studies have shown the ability of this marker to differentiate between *S. scrofa* clades^55,58^ but has not been used in the context of wild suid populations. The nuclear marker *MC1R* plays a key role in regulating eumelanin (black/brown) and phaemelanin (red/yellow) and is responsible for coat, hair, and skin colour variation in various pig breeds^48,59^ and may be useful in distinguishing the presence of population differences based on coat colour. Partial genes from each of the MHC class I, II and III were also sequenced to assess the immunogenetic diversity of bushpigs from Madagascar. The MHC class I classical genes were not targeted due to their high level of gene duplication which can reduce the resolution of analyses. Instead, the MHC class I non-classical *SLA-6* exon-2 region was targeted (alpha-1), as well as *SLA-DQB1* exon-2 (class II), and region spanning residues 450–518 (exons 11–14) of the class III *BAG6* gene, which has been suggested to be associated with ASFV infection in the host^60,61^. Primers used for sequencing the MHC class I and II genes were designed based on the *S. scrofa* haplotype Hp1a.1^31,62,63^ and primers class III *BAG6* were designed by the Australian Genome Research Facility (AGRF) Ltd, Australia, based on the Sscrofa11.1 reference genome (Gene ID: 100153950) (Table 2). PCR and sequencing were performed at AGRF Ltd, Australia. The raw forward and reverse chromatograms were assessed, and sequences manually edited using SeqTrace^64^. Sequences were then assembled to give a consensus sequence for each sample (excluding primer sequences).

**Table 2:** Oligonucleotide primers used to amplify and sequence mitochondrial (mtDNA) and nuclear DNA sequences (nuDNA).

Genomic region	Locus	Primer name	Primer sequence (5′ → 3′)	Target size (bp)	Source
mtDNA	CR	CR L CR H	CCAAGACTCAAGGAAGGAGA GGCGCGGATACTTGCATGTG	1036	^46^
	*cytb*	LT4724 H15915R	CGAAGCTTGATATGAAAAACCATGGTTG GGAATTCATCTCTCCGGTTTACAAGAC	1140	^47^
nuDNA	*GPIP*	GPIP1 GPIP6	TGCAGTTGAGAAGGACTTTACTT GAAGTTACAGGGCATCATCTTG	507	^48,49^
	*MC1R*	MC1R-F MC1R-R	AGTGCCTGGAGGTGTCCATTCAC CGTAGATGAGGGGGTCCAGGATAGA	795	^50^
	*SLA-6 *(class I)	SLA-6F SLA-6R	TCAGCCYCTCCCTGTTMTAG GTTCCTGCACCCCCTTASAC	314	Lee et al., unpublished
	*SLA-DQB1 *(class II)	DQB1-e2_F DQB1-e2_R	GCCTGACTGACGCGGTATCTC GAGTGCCTGCCCGCC	315	Lee et al., unpublished
	*BAG6 *(class III)	BAG6-F BAG6-R	CCCTTGCTCCCTCTTCTACC GCTTCTCATGCAGCCTGTG	895	This study

### Datasets used for downstream analyses

The number and assignment of haplotypes for CR, *cytb*, *GPIP* and *MC1R* were determined using DnaSP v6^65^. To determine the phylogenetic position of bushpigs from Madagascar in relation to other members of the family Suidae, we also included available sequences of the CR, *cytb*, *GPIP*, and *MC1R* from NCBI (https://www.ncbi.nlm.nih.gov/) for wild species of pigs and peccaries (Supplementary Table S2)^48,66–73^. The following Datasets were prepared for downstream analyses (Table 3). Dataset (1a) includes concatenation of both mitochondrial sequences (CR, *cytb*) from this study and available NCBI data; (1b) includes either mtDNA sequences available for each species/sample; (2) sequenced and available NCBI *GPIP* region; (3) sequenced and available NCBI *MC1R* region; (4) *SLA-6*; (5) *SLA-DQB;1* and (6) *BAG6* sequences from this study. For Datasets 1a and 1b, sequences were aligned in ClustalW^74^ and concatenated in SequenceMatrix^75^ to produce one sequence for each representative species. Concatenated sequences are useful to overcome sampling error and missing data, and can show accuracy in generating taxonomic topologies^76–78^.

**Table 3:** Datasets used for phylogenetic and/or haplotype analyses.

Dataset no	Gene	Number of sequences		Partition/model	Aligned length (bp)	Concatenated length (bp)
		Bushpigs	Other suids and tayassuids			
1a	CR	98 (1)	12 (20)	HKY + I + G	1451	2592
	*cytb*	110 (1)	14 (17)	HKY + G	1141	
1b	CR	110 (1)	16 (20)	HKY + I + G	1451	2592
	*cytb*			HKY + G	1141	
2	*GPIP*	102 (1)	14 (11)	K80 + I	462	
3	*MC1R*	100 (0)	14 (4)	HKY + I	787	
4	*SLA-6*	104	16	K2	270	
5	*SLA-DQB1*	94	16	T92	270	
6	*BAG6*	96	34	JC	433	

The gene, number of sequences, aligned length, and concatenated length of each dataset are indicated. The partition and model for phylogenetic and genetic diversity analyses are indicated for the respective genes. Numbers in brackets indicate the number of NCBI sequences used. Accession numbers are provided in Supplementary Table S2.

### Inferring the relationship of bushpigs in Madagascar

Phylogenetic inferences were generated based on Datasets 1–3 (Table 3) using the RAxML-NG v0.09.0 web-based server^79^. The partitions to accommodate for different evolutionary models were based on the Bayesian Information Criterion^80^ identified by ModelGenerator^81^ and listed for each gene in Table 3. PopArt v1.7^82^ was used to produce Medium-Joining Networks (MJN) of only bushpig sequences to visualise the intra-relationships of mainland and Madagascar samples of different loci in this study (Datasets 1–6).

### Analyses of MHC genetic diversity and selection

To observe whether the MHC sequence of bushpigs from Madagascar has been subjected to purifying or diversifying selection since their introduction to the island, we performed selection analysis using DataMonkey^83^. We estimated selection based on (1) all haplotypes identified, (2) bushpigs from Madagascar only, and (3) mainland bushpigs—selection for *SLA-6* was based on all haplotypes only as three or more input sequences in DataMonkey is required. Selection tests can be used for identifying certain positions in genes or regions (particularly in coding regions) that are conserved or have underwent radical changes in some taxa, and for testing an increase of nonsynonymous substitutions than expected under neutral evolution^84^. Diversifying selection is observed by a higher nonsynonymous substitution rate (dN) compared to the synonymous substitution rate (dS). A dN/dS ratio > 1 suggests the presence of positive or diversifying selection; dN/dS < 1 indicates negative or purifying selection; and dN/dS ~ 1 indicates no selection. We tested four different methods for detecting sites under selection: MEME (Mixed Effects Model of Evolution), FEL (Fixed Effects Likelihood), SLAC (Single-Likelihood Ancestor Counting), and REL (Random Effects Likelihood). For each method, the significant levels were the following: *P* ≤ 0.05 in MEME^85^, *P* ≤ 0.25 in FEL and SLAC, and a Bayes Factor of > 50 in REL^86^. Only sites significantly identified under positive selection by at least two methods were considered^87,88^.

Genetic diversity was calculated in MEGA 7^89^ using the model-of-best-fit Tamura 92 (T92)^90^, Kimura-2 parameter (K2)^91^, and Jukes-Cantor (KC)^92^ for *SLA-DQB1*, *SLA-6,* and *BAG6,* respectively. Standard error was calculated using 1,000 bootstraps. Based on initial genetic diversity analysis, genetic distance was also calculated for *SLA-DQB1* in MEGA 7 using p-distance in coding positions only and sites with < 95% coverage were discarded from analysis.

### Divergence of bushpigs from mainland Africa and Madagascar

BEAST v2.4.8^93^ was used to estimate the divergence time between bushpigs in mainland Africa and Madagascar. Concatenated mitochondrial CR and *cytb* sequence alignments were subsampled to include members of Potamochoerini, Phacochoerini, and Suini, a *Babyrousa*, and a *Pecari* as an outgroup (Dataset 1a; Table 3; Supplementary Table S1). Independent test runs using the *MC1R* and *GPIP* nuclear genes were also performed and evaluated. The sequences were aligned using ClustalW algorithm in Geneious v.11.0.4 (https://www.geneious.com)^94^. PartitionFinder2 on XSEDE^95^ was used to determine the best substitution model and best partitioning scheme for the dataset. It provided four data blocks including the CR, and the first, second, and third codon of the *cytb* gene (Supplementary Table S3). The best partitioning scheme and substitution model as indicated by PartitionFinder were used in the downstream BEAST analysis. BEAST was implemented using a calibrated Yule tree prior and a single uncorrelated relaxed lognormal clock model. The molecular clock was calibrated by constraining the time to most recent common ancestor (TMRCA) of the Suoidea (Tayassuidae-Suidae split) according to normal distribution prior with a mean of 37.09 Mya and a sigma of 0.5. The Suiodea TMRCA prior around of 34.50–39.69 Mya was previously estimated and is in consensus with other estimated molecular and fossil analyses^67^. The BEAST MCMC chain was set to run for 20 million generations (replicated three times) with the first 10% set as burn-in. The models and parameters for the BEAST run were prepared using BEAUTi^96^. The BEAST analysis was independently replicated three times using a different starting seed each time. All the generated log and tree files were combined using LogCombiner^97^. The BEAST analysis was evaluated using Tracer v1.7.1^98^ by ensuring an effective sample size (ESS) of > 200 and convergence. TreeAnnotator^97^ was used to summarize the tree topologies, generate the best supported BEAST trees (maximum clade credibility tree) corresponding to the 95% HPD ranges, and to estimate the posterior clade probability of each node. FigTree v.1.4.3 (https://tree.bio.ed.ac.uk/software/figtree/)^99^ was used to visualize and evaluate the estimated tree.

### Ethics approval and consent to participate

Bushpig samples were collected under permission from the Direction Générale des Eaux et Forêts de Madagascar or were collected from hunters, and museum specimens were provided by the Field Museum of Natural History, Illinois, USA. Other species were sourced from previous studies^31,62,63^.

## Results

### Phylogenetic relationship and divergence of bushpigs from Madagascar to other Suidae

**Figure 2:**
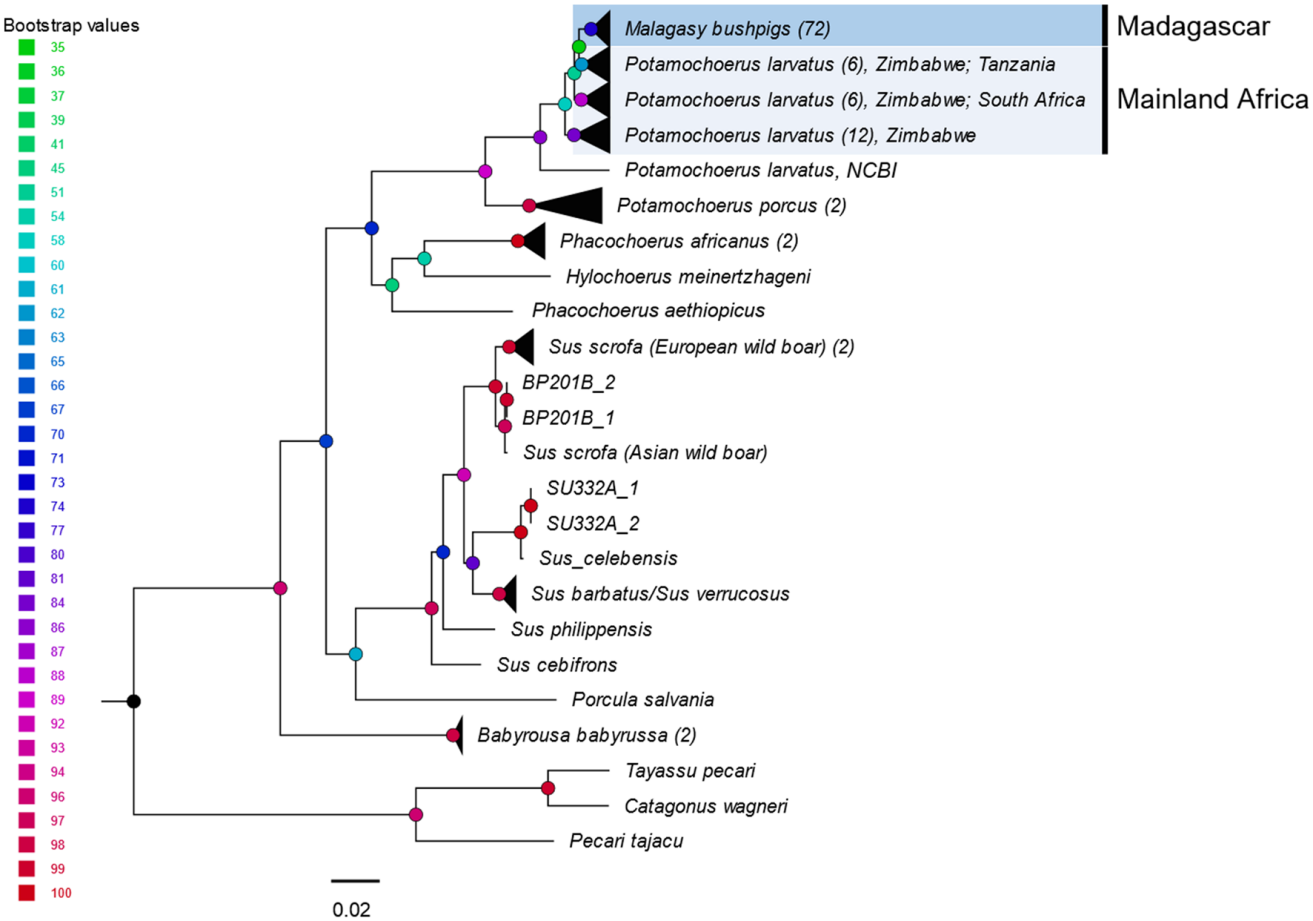
Phylogenetic tree indicating the position of Malagasy bushpigs as *Potamochoerus larvatus*. A Maximum Likelihood tree was generated, based on concatenated mtDNA (CR and *Cytb*) and nuDNA (*GPIP* and *MC1R*) sequences, using the RAxML-NG v0.6.0 web-server^79^ (https://raxml-ng.vital-it.ch/#/). Statistical support was assessed using a bootstrap cut-off of 0.03 (Bootstrap support indicated above the branches). Numbers in brackets indicate the number of sequences from this study within each node.

Phylogenetic analysis of concatenated mtDNA (Dataset 1a; Fig. 2) and non-concatenated mtDNA genes (Datasets 2–3; Supplementary Fig. S1 and Fig. S2) using the maximum likelihood method showed that specimens of bushpigs from Madagascar and mainland bushpigs formed a monophyletic clade. Bushpigs from “East”, “West” or “North” Madagascar, did not form reciprocal monophylogetic lineages. Furthermore, some mainland bushpigs, including those from Zimbabwe (*n* = 3) and Tanzania (*n* = 1) appear as a sister clade to Madagascar bushpigs. This is followed by those from South Africa (*n* = 2), and the remaining specimens from Zimbabwe (*n* = 6). Trees inferred from Dataset 1b (Supplementary Fig. S3) was consistent to Dataset 1a. The topology for nuclear DNA markers was less resolved. Both *GPIP* and *MC1R* showed little resolution of bushpig samples but were sufficient to cluster other extant suids separately from bushpigs (Supplementary Fig. S3 and Fig. S4). Surprisingly, one bushpig sample obtained from Tervuren (Belgium) clustered with *S. scrofa* samples for each analysis; presumably due to sampling error/mislabelling. The specimens of bushpigs from Madagascar employed herein are genetically distinct, even in the highly conserved *GPIP* and *MC1R* genes, from the *P. porcus* specimens available for this study. The 56 CR, 62 *cytb*, 52 *GPIP*, and 51 *MC1R* have been submitted to GenBank under accession numbers: MT853484–MT853538, MT864081–MT864142, MT864030–MT864080, and MT864143–MT864192.

Based on the concatenated mitochondrial DNA dataset, the BEAST analysis places the node age estimates for the divergence of Malagasy bushpigs and mainland African bushpigs at 0.49 MA (node A; 95% Higher Posterior Density [HPD] 0.27–0.77 Ma). The Bayesian Posterior Probability (BPP) score for this node was 0.73. Time divergence for the split between other mainland African bushpigs (nodes B and C) was estimated to be between 0.41 and 0.76 Ma (BPP = 1) and the split from *P. porcus* estimated at 1.81–4.34 Ma (node D). The TMRCA of the Suidae group was 7.74 Ma (95% HPD 5.42–10.44 Mya; BPP = 0.82), while the split between Suinae and Babyrousinae is at 9.27 Mya (95% HPD 5.94–12.68; BPP = 0.99). None of the nodes got a BPP score of < 0.5.

We did not estimate divergence time between Madagascan and mainland African bushpigs using the nuclear gene *MC1R* and pseudogene *GPIP* as test runs produced very unresolved trees. In addition, previous work did not include the *MC1R* gene for divergence time estimation due to strong phenotypic selection^48^.

### Haplotype structure of bushpigs from Madagascar

**Figure 3:**
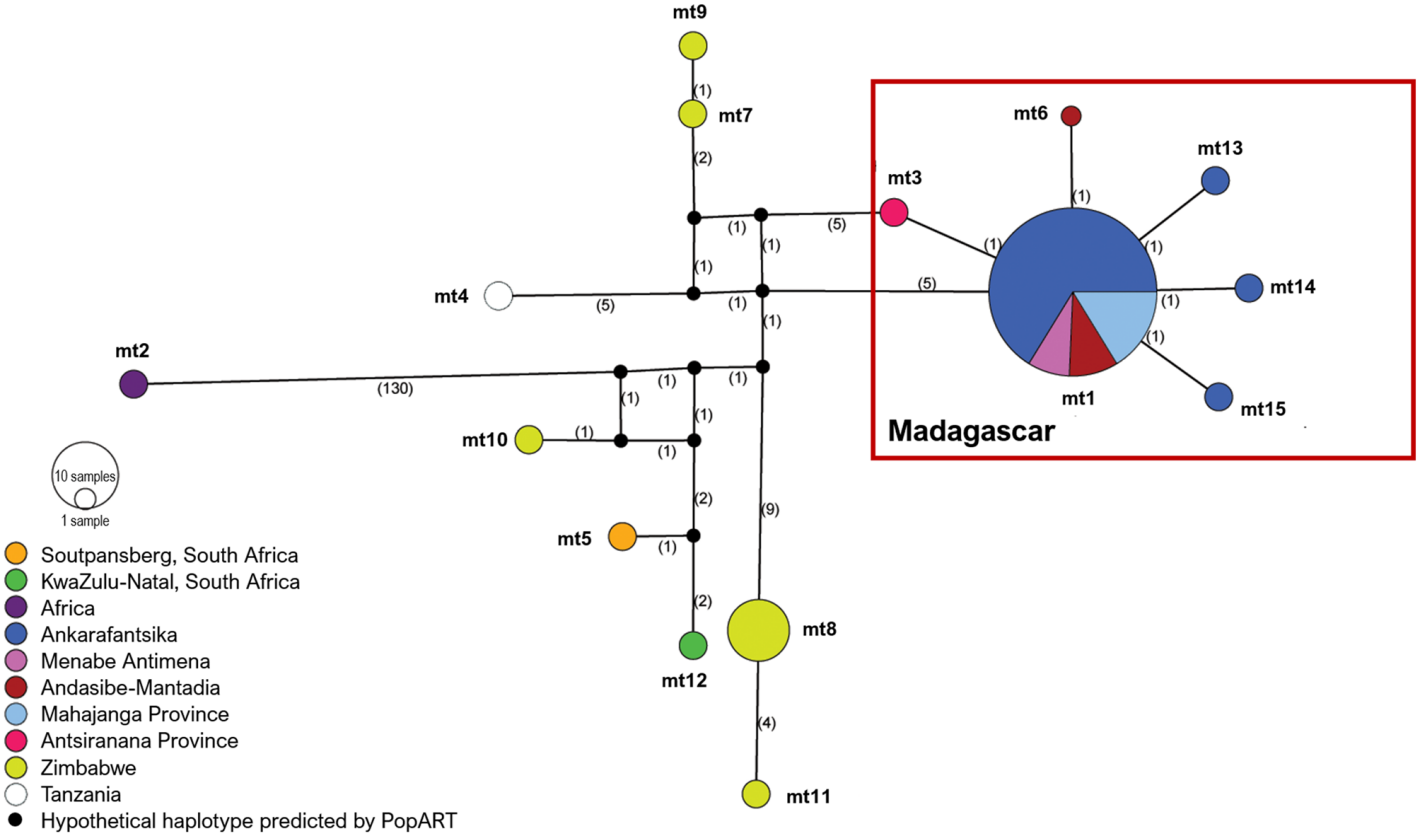
Median joining network of Malagasy bushpigs based on concatenated mtDNA (CR and *cytb*). Coloured circles represent the haplotypes identified by DnaSP v6^65^ with each colour represented by a location as indicated by the figure key, and the size of the circle is proportional to the frequency of each haplotype (with ‘mt’ as a prefix to indicate mitochondrial). Numbers in parentheses along the branches represents the mutational steps between each haplotype.

**Figure 4:**
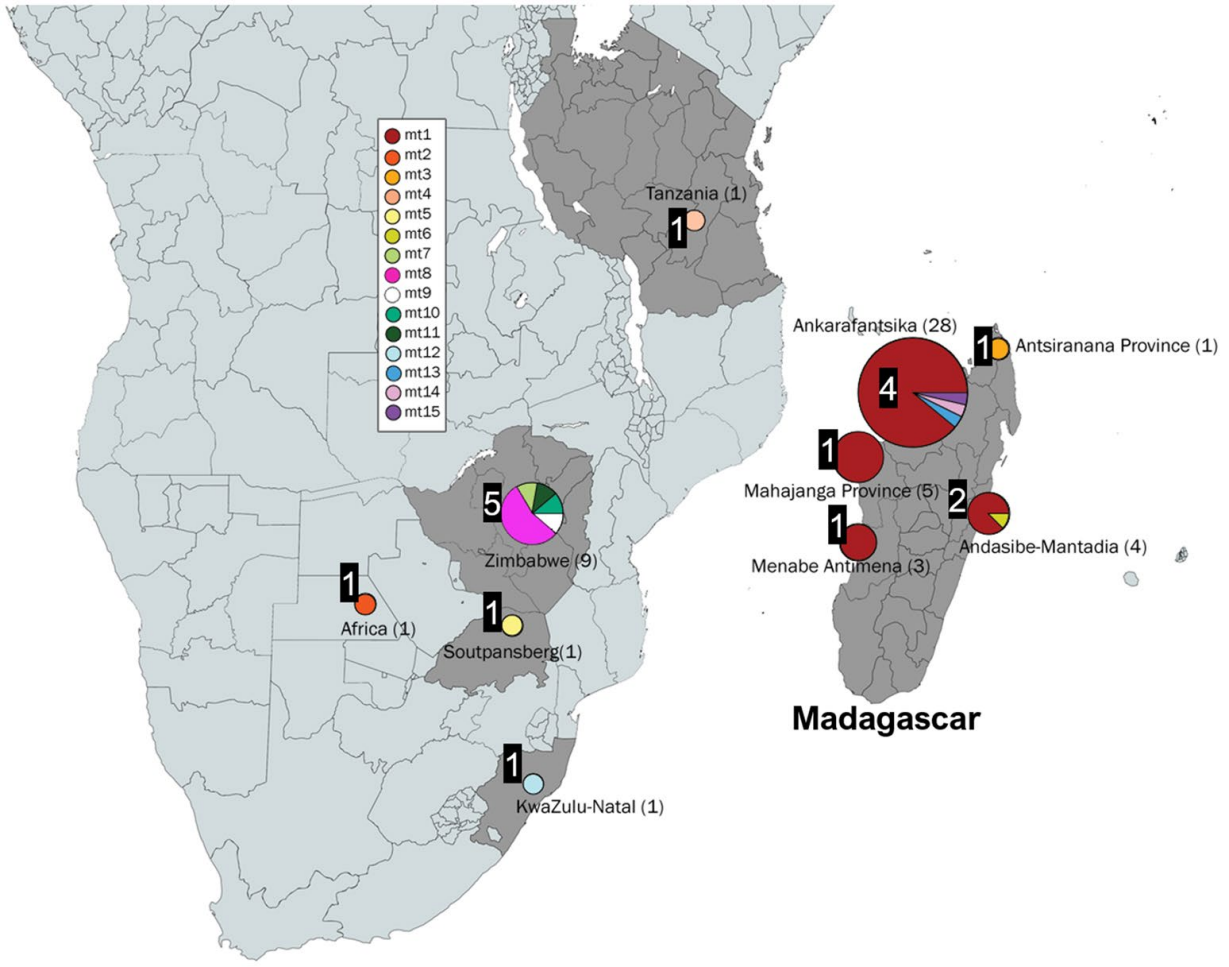
Malagasy bushpig mtDNA (CR and c*ytb*) sequence by sampling location. Each colour indicates the different haplotypes (with ‘mt’ as a prefix to indicate mitochondrial) with the number of individuals in each population shown in brackets. The number of different haplotypes within each location is highlighted by the black box adjacent to each circle. Figure includes an edited map generated by mapchart.net and haplotypes produced in PopART^82^ (https://popart.otago.ac.nz/index.shtml).

Haplotypes identified by DnaSP were assigned the prefixes ‘mt’ (concatenated mitochondrial DNA from Datasets 1a and 1b), ‘CR’ (control region), ‘*cytb’ (cytochrome b*), ‘GPIP’, and ‘MC1R’ followed by a designated number. MJN analysis of concatenated bushpig mtDNA sequences (Dataset 1b; Table 3) showed that the haplotypes of bushpigs from Madagascar clustered separately from mainland bushpigs (Figs. 3 and 4). A total of six closely related haplotypes were found from the concatenated data (Dataset 1b; Fig. 3); one haplotype (mt1) shared by Madagascar bushpigs from the Mahajanga Province in West-Northwest (Menabe Antimena, Ankarafantsika, and general Mahajanga Province), three unique haplotypes found in bushpigs from Ankarafantsika (mt13, 14, and 15), and one each from Andasibe-Mantadia (East Madagascar; mt6), and the Antsiranana Province (North Madagascar; mt3). Single-gene analysis of the CR and *cytb* (Supplementary Fig. S6 and Fig. S7) showed no shared haplotypes between bushpigs from Madagascar and mainland Africa. Both CR and c*ytb* contributed the most to the genetic differentiation between these two groups (13 and 14 haplotypes, respectively) each with five haplotypes unique to Madagascar. In contrast, the nuclear *GPIP* and *MC1R* genes (Supplementary Fig. S8) were highly conserved between bushpigs showing respectively three (two found in Madagascar) and four haplotypes (three found in Madagascar), and provided the lowest resolution of bushpig relationships.

The above was supported by concatenated sequences in which both mtDNA genes were successfully sequenced (Dataset 1a; Table 3), showing that specimens from Madagascar clustered closely together and separately from mainland specimens (Supplementary Fig. S9). In this case, nine haplotypes were identified for bushpigs from Madagascar, and of these, four haplotypes here were classified as mt1 using Dataset 1b (Fig. 3). Two of these haplotypes were found only in the Ankarafantsika specimens and the general Mahajanga Province region. Based on MJN analysis which showed the close relatedness of specimens from across Madagascar (one mutational step between haplotypes), the occurrence of multiple bushpig introductions to the island is unlikely.

Regarding mainland bushpigs, based on Datasets 1a and 1b, five unique haplotypes (mt7, 8, 9, 10, and 11) were identified in Zimbabwe bushpigs and were more distantly related to each other compared to bushpigs from Madagascar (higher number of substitutions; Fig. 3 and Supplementary Fig. S9). The Zimbabwe bushpgis were also genetically different from bushpigs sampled from the Soutpansberg (mt5), KwaZulu-Natal (mt12), and Tanzania (mt4). The Soutpansberg (mt5) and KwaZulu-Natal (mt12) bushpigs were consistently grouped separately from other bushpigs from Madagascar in all other mtDNA analyses (Supplementary Fig. S6 and Fig. S7). These results suggest that bushpigs from the island are related to the specimens from the central region of southern Africa. However, the absence of genetic data from southern Tanzania and Mozambique also limits the resolution of the relatedness between bushpigs from other regions in mainland Africa and Madagascar.

Overall, based on Dataset 1, the most frequent haplotype was mt1 found in 91.5% of bushpigs from Madagascar (Table 4). It was mostly found in specimens from “West” Madagascar (90.5%), where mt13-15 were also found at low frequencies (2.7–3.6%).

**Table 4:** Percentage of CR and c*ytb* concatenated haplotypes found in bushpigs across Madagascar and mainland Africa.

Haplotype prefix	*N*	mt														
Location		1	2	3	4	5	6	7	8	9	10	11	12	13	14	15
**Madagascar**	42	88.1		2.4			1.2							2.4	2.4	2.4
“West” Madagascar	37	90.5												2.7	2.7	2.7
Ankarafantsika	28	87.5												3.6	3.6	3.6
Menabe Antimena	3	100														
Mahajanga Province general	6	100														
“East” Madagascar (Andasibe-Mantadia)	4	87.5					12.5									
“North” Madagascar (Antsiranana Province)	1			100												
**Mainland Africa**	13		7.7		7.7	7.7		7.7	38.5	7.7	7.7	7.7	7.7			
Tanzania	1				100											
Zimbabwe	9							11.1	55.6	11.1	11.1	11.1				
KwaZulu-Natal, SA	1												100			
Soutpansberg, SA	1					100										
Africa	1		100													
**Total (All bushpigs)**	54	68.5	1.9	1.9	1.9	1.9	0.9	1.9	9.3	1.9	1.9	1.9	1.9	1.9	1.9	1.9

Percentages are based on the number of samples for each location. Haplotypes correspond to those labelled from Dataset 1a (Fig. 3).

### Haplotype structure and genetic diversity of three MHC loci

**Figure 5:**
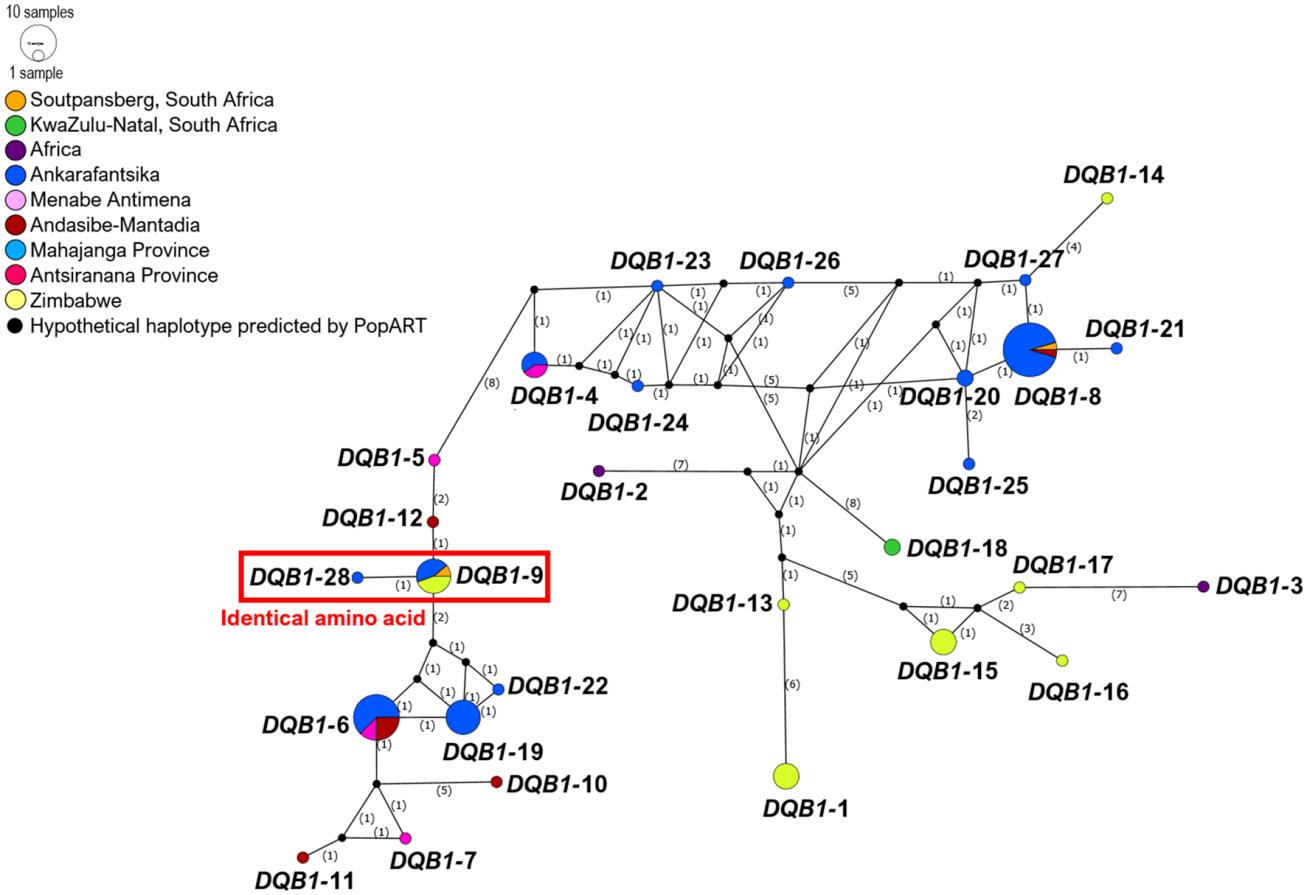
Median joining network of Malagasy bushpigs based on MHC loci *SLA-DQB1* exon-2 sequence. Coloured circles represent the haplotypes identified by DnaSP v6^65^ with each colour represented by a location as indicated by the figure key, and the size of the circle is proportional to the frequency of each haplotype. Numbers in parentheses along the branches represents the mutational steps between each haplotype. Haplotypes with the same translated amino acid sequence are outlined.

**Figure 6:**
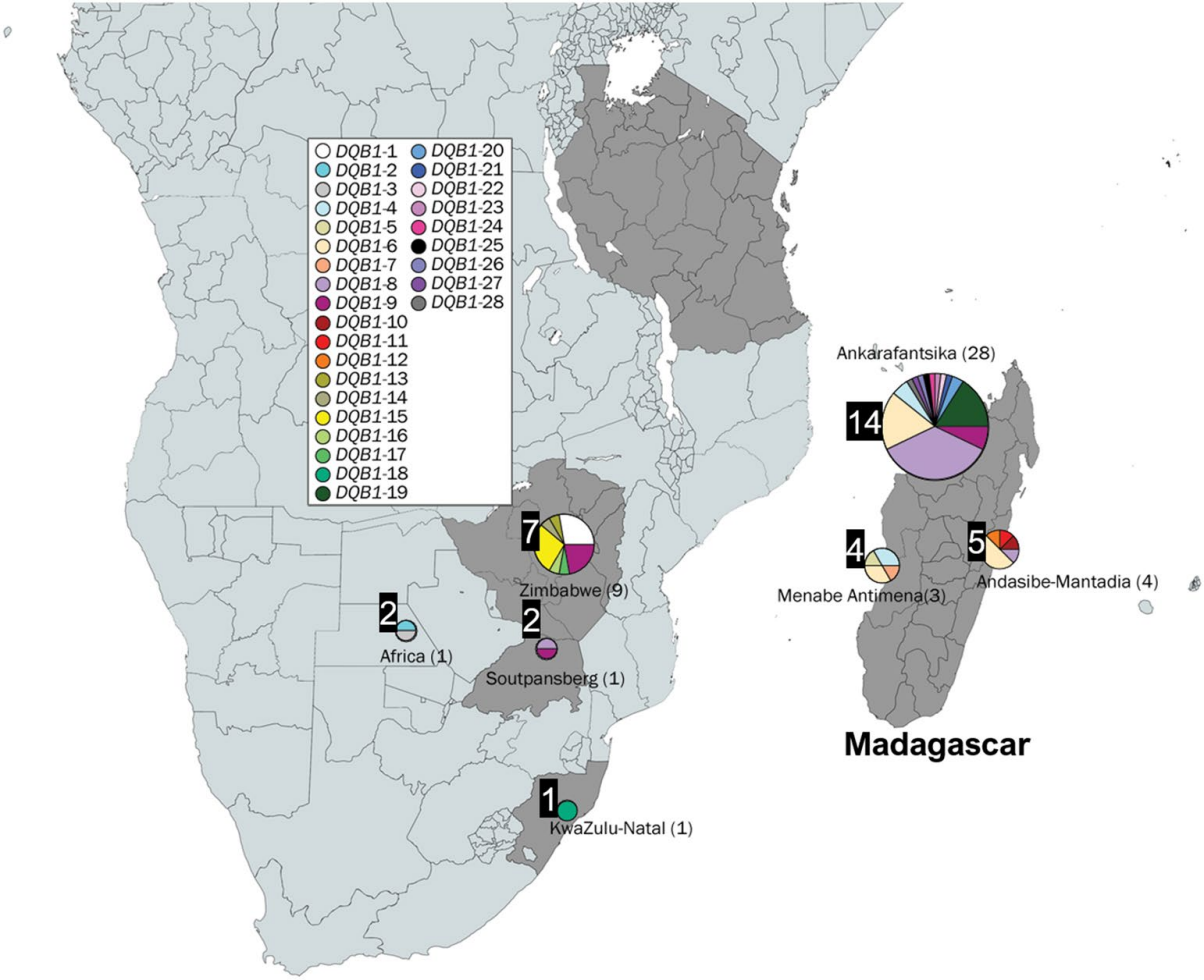
Malagasy bushpig MHC class II *SLA-DQB1* sequences by sampling location. Each colour indicates the different haplotypes with the number of individuals in each population shown in brackets. The number of different haplotypes within each location is highlighted by the black box adjacent to each circle. Figure includes an edited map generated by mapchart.net and haplotypes produced in PopART^82^ (https://popart.otago.ac.nz/index.shtml).

**Figure 7:**
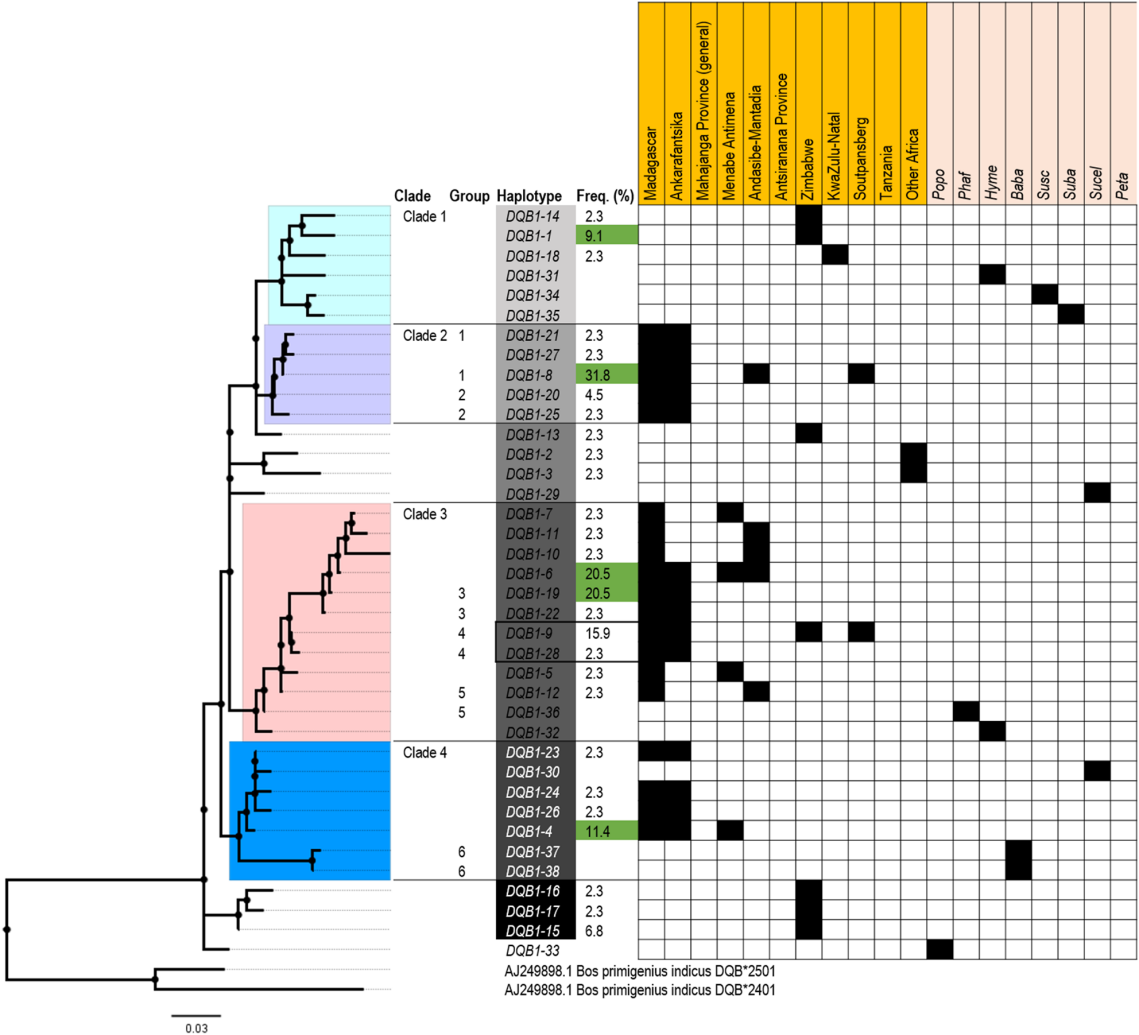
Phylogenetic analysis of the *SLA-DQB1* haplotypes including all haplotypes (and samples). Haplotype numbers are aligned to their position on the maximum likelihood tree with their frequencies (%). The presence of each haplotype in the specific localities is indicated by the shaded box. Group numbers indicate identical antigen-binding sites of the relevant haplotypes.

Median-Joining Network analysis found no specific relationship or clustering for *SLA-DQB1* exon-2 sequences (Figs. 5 and 6). However, phylogenetic analysis suggests that certain haplotypes clustering together in the same clade may perform similar functions (as supertypes) due to shared antigen-binding sites. Most of these were shared within bushpigs (Fig. 7; groups 1, 2, 3, and 4), while group 5 was shared between bushpigs and *P. africanus,* and group 6 was found in *B. babyrussa* only. Antigen-binding site Groups 2 and 5 were found in relatively low frequencies (average 3.4% and 2.3%, respectively).

The highest genetic diversity (nucleotide; nt: 0.048; amino acid; AA: 0.117) was observed in the polymorphic *SLA-DQB1* exon-2 region (Table 5) with 28 haplotypes—19 in bushpigs from Madagascar and 11 in mainland individuals (Supplementary Table S4). Only two haplotypes were shared between mainland and bushpigs from the island (*DQB1-*8 and *DQB1-*9). The most frequent of these was *DQB1-*8 (31.8% of all bushpig specimens) which was found in specimens from Ankarafantsika (42.9%), “East” Madagascar from Andasibe-Mantadia (25%) and Soutpansberg on mainland Africa (Fig. 6; Supplementary Table S4). *DQB1-*4, -6, and -19 were also found in relatively high frequencies in Madagascar (11.4–20.5%; Supplementary Table S4). The remaining 15 haplotypes were found in low frequencies (2.3–9.1%), mostly from Ankarafantsika. The genetic diversity between Madagascar and mainland bushpigs was similar (nt: 0.043; amino acid; AA: 0.114 and mainland Africa nt: 0.046; AA: 0.098), although diversity appears lower when looking at specific sampling locations separately in Madagascar (Supplementary Table S4). Amongst the 28 haplotypes found in bushpigs, only one (*DQB1-*15; 6.8%) was shared with another species (*P. porcus*). No existing sequence was found on the NBCI and IPD-MHC database (Supplementary Table S5).

**Table 5:** Diversity analysis of bushpigs MHC class II *SLA-DQB1* exon-2 sequence.

	nhap	nt			AA		
		V	mean	S.E	V	MEAN	S.E
**All**	38	43	0.048	0.008	28	0.117	0.023
**Bushpigs**	28	31	0.046	0.008	21	0.115	0.025
**Madagascar**	19	27	0.043	0.009	20	0.114	0.027
Ankarafantsika	14	19	0.038	0.009	16	0.101	0.025
Mahajanga Province (general)	–						
Menabe Antimena	4	14	0.031	0.008	13	0.09	0.025
“East” Madagascar (Andasibe-Mantadia)	5	19	0.039	0.009	16	0.104	0.027
“North” Madagascar (Antsiranana Province)	–						
**Mainland Africa**	11	28	0.046	0.009	18	0.098	0.024
Zimbabwe	7	26	0.042	0.008	18	0.093	0.022
KwaZulu-Natal, SA	1						
Soutpansberg, SA	2						
Tanzania	–	13	0.051	0.013	10	0.121	0.028
Africa	2	9	0.035	0.012	7	0.083	0.032

Analysis was performed using the model T92 in MEGA 7^89^. The Poisson model was used for amino acid analysis. The number of haplotypes (nhap) for each category is shown, including mean diversity (and standard error; S.E) and variable sites (V) for nucleotide (nt) and amino acid (AA) sequences.

Genetic distance analyses indicated that the haplotypes are also very distinct from those found on the mainland; genetic distance between Madagascar and mainland bushpig sequences ranged from 0.031 to 0.058, while sequences from Madagascar (Ankarafantsika, Menabe Antimena, and Andasibe-Mantadia) had a genetic distance ranging from 0.026 to 0.035 (Supplementary Table S6).

In contrast to *SLA-DQB1*, the class II *SLA-6* and class III *BAG6* genes were more conserved with only two and four haplotypes found, respectively. For both genes, only one haplotype was found in Madagascar (*SLA-6*-1 and *BAG6*-1; Supplementary Fig. S10 and Fig. S11), which was similarly found in high frequencies on mainland Africa as well (91.7–100% in *SLA-6* and 83.3–92.3% in *BAG6*; Supplementary Table S5 and Fig. S6). Interestingly, these were also found in other sub-Saharan African species (*P. porcus, H. meinertzhageni,* and *P. africanus*) and was shared with *B. babyrussa* through identical amino acid translation (*SLA-6*-7). Within *BAG6* (Supplementary Table S7), *BAG6-*3 to 5 were found in lower frequencies but the aforementioned and *BAG6-*6 produced the same amino acid sequence as the most common haplotype (*BAG6-*1) and thus appears fairly conserved across mainland and Madagascar bushpigs and not found in other species. Based on amino acid translation, haplotypes *BAG6*-1 and *BAG6*-6 were found in other sub-Saharan African species and *B. babyrousa*, respectively. *BAG6*-7 and *BAG6*-8 were only found in *P. tajacu.* The overall genetic diversity of *SLA-6* was lower (nt: 0.03; AA: 0.04; Table 6) than *BAG6* (nt: 0.017; AA: 0.01; Table 6), although the diversity was lower in *BAG6* when only considering bushpigs (nt: 0.004; AA: 0.004). However, the S.E. for both these genes was high, possibly due to the low number of haplotypes found. In addition, the number of variable sites was extremely low. BLAST searches also found three identical matches in public databases, with *SLA-6-*2 matching several *SLA-6* haplogroups of *S. scrofa*, *SLA-6*-4 being identical to SLA-6*10:01 (Supplementary Table S9)*,* and *BAG6-*2 being identical to an existing *S. scrofa* sequence (Supplementary Table S10). These sequences were also shared with other *Sus* species. The 86 *SLA-DQB1,* 74 *SLA-6*, and 65 *BAG6* sequences were submitted under GenBank accession numbers: MT853335–MT853419, MT853262–MT853334, and MT853420–MT853483.

**Table 6:** Diversity analysis of bushpigs MHC class I *SLA-6* exon-2 and class III *BAG6* loci.

	*SLA6*							*BAG6*						
	nhap	nt			AA			nhap	nt			AA		
		V	Mean	S.E	V	Mean	S.E		V	Mean	S.E	V	Mean	S.E
**All**	7	17	0.03	0.007	7	0.04	0.015	8	17	0.017	0.004	4	0.011	0.006
**Bushpig**	2	2	0.007	0.005	1	0.011	0.011	4	3	0.004	0.002	1	0.004	0.004
**Madagascar**	1							1						
Ankarafantsika	1							1						
Mahajanga Province (general)	1							1						
Menabe Antimena	1							1						
“East” Madagascar (Andasibe-Mantadia)	1							1						
“North” Madagascar (Antsiranana Province)	1							–						
**Mainland Africa**	2	2	0.007	0.005	1	0.011	0.011	4	3	0.004	0.002	1	0.004	0.004
Zimbabwe	1							3	2	0.004	0.002	1	0.005	0.005
KwaZulu-Natal, SA	1							1						
Soutpansberg, SA	1							1						
Tanzania	1							–						
Africa	1							1						

MEGA 7^89^ was used to perform genetic diversity analysis for *SLA-6* and *BAG6* based on the models K2 and JC, respectively. The Poisson model was used for amino acid analysis. The number of haplotypes (nhap) for each category is shown, including mean diversity (and standard error; S.E.) and variable sites (V) for nucleotide (nt) and amino acid (AA) sequences.

### Selection analysis of MHC in bushpigs from Madagascar

**Figure 8:**
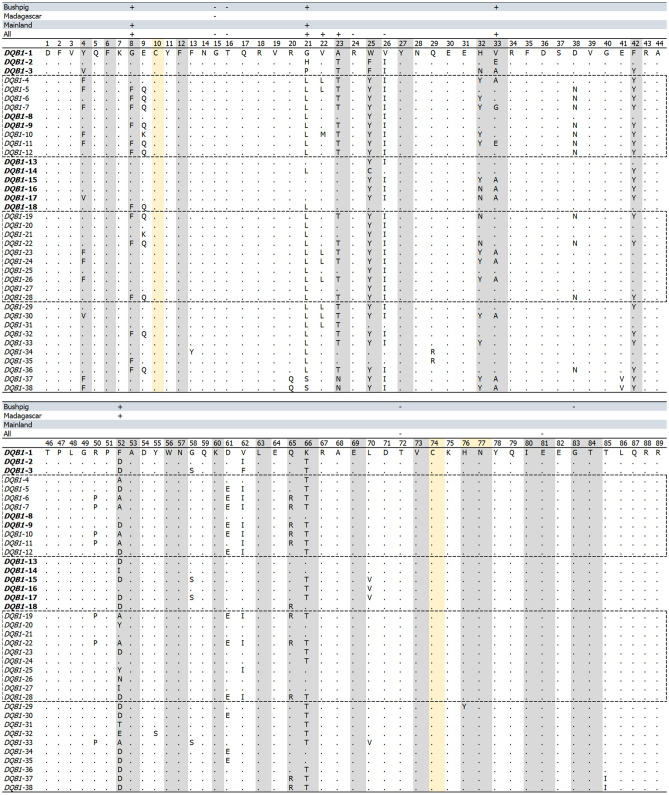
Amino-acid sequence and selection of *SLA-DQB1* exon-2 haplotypes. Analysis was completed using DataMonkey HyPhy server^83^. Dots represent identical residues to *DQB1*-1 at the positions shown. Grey shaded sites indicate the antigen-binding sites and yellow indicate the conserved cysteines. Positions significant for positive (+) or negative (−) selection for at least two methods (MEME/FEL/SLAC/REL) are indicated for each population/location category. Dotted lines indicate haplotypes found in bushpigs and haplotypes in bold font are found in mainland bushpigs.

Several *SLA-DQB1* sites were found to be under (diversifying) selection when considering all haplotypes or subcategories (mainland Africa or Madagascar; Fig. 8). Two and one sites were detected under diversifying selection respectively in mainland bushpigs (8G: MEME *P-*value: 0.03, REL BF: 208.31; 21G: MEME *P-*value: 0.03, SLAC *P-*value: 0.13, REL BF: > 50), and bushpigs from Madagascar (52F: FEL *P-*value: 0.16, SLAC *P-*value: 0.16). Site 21 was the most diverse in mainland bushpigs, coding for up to four different residues, the same site only coding for one residue in bushpigs from Madagascar. In contrast, site 52 coded for up to five different residues in bushpigs from Madagascar, compared to only three in mainland bushpigs (Fig. 8). In addition, one site was significant for purifying selection in bushpigs from Madagascar (15G: FEL *P-*value: 0.06, SLAC *P-*value: 0.13, REL BF: > 50), and was conserved across all haplotypes.

Results show that the *SLA-6* exon-2 region was highly conserved (Supplementary Fig. S12), with two sites subjected to purifying selection in more than two methods (42 N: FEL *P-*value: 0.07, SLAC *P-value:* 0.25; and 65D: FEL *P-*value: 0.09, SLAC *P-value:* 0.25) and one significant for diversifying selection (57R: FEL *P-*value: 0.25, MEME: 0.23, REL Bayes factor*:* 346.4). Site 57 coded for two different residues (arginine or serine) but was conserved in bushpig haplotypes (Supplementary Fig. S12). *BAG6* was similarly conserved (Supplementary Fig. S13); when all haplotypes were considered, we detected one site evolving under purifying selection (26I: FEL *P-*value: 0.04, SLAC *P-*value: 0.17) and three diversifying selected sites (47P: FEL *P-*value: 0.20, REL BF: 98.71; 94P: FEL *P-*value: 0.07, MEME *P-*value: 0.04, REL BF: 9478.98; and 113A: FEL *P-*value: 0.24, REL BF: 90.14). Sites 47, 94 and 113 coded for two different residues each. No difference between mainland bushpigs and those from Madagascar were found for both *SLA-6* and *BAG6*.

## Discussion

### Presence of bushpigs in Madagascar

The geographic origin of bushpigs, from the genus *Potamochoerus,* in Madagascar has been speculated for a considerable time—whether they were introduced and if so, from what area of Africa. Molecular evidence identified that bushpigs on the island are derived from the sub-Saharan Africa species, *P. larvatus* as a sister clade of *P. porcus*^67^ and the Madagascar population originated from a lineage that is closest to the populations of the central region of southern Africa, perhaps related to the subspecies *P. l. koiropotamus* found predominantly in southern Africa^15^. The Mozambique Channel may have had formed a barrier to the direct passage of seafaring people from coastal Africa direct to Madagascar and this passage may have been more easily navigated via the Comoro Islands^21^; the later passage was an important trade route for sailing vessels for at least a portion of the Middle Age^100^.

As all Malagasy samples formed a well-supported monophyletic clade (Fig. 3 and Supplementary Fig. S9), our results do not support the idea that bushpigs in Madagascar originated from multiple African source populations. It remains possible, however, that additional haplotypes could have been identified with a greater geographical sampling, particularly in the far north (Antsiranana Province) and also the Comoro Islands (i.e., Grande Comore and Mayotte^9,101^)—but evidence of bushpigs on this island are unverified reports and may have been mistaken for feral pigs^101^. BEAST analysis suggests that Malagasy bushpigs diverged from an African source population, that was closest to bushpigs from Zimbabwe, approximately 480,000 years ago. These populations are distinct from those other Zimbabwe specimens and those in KwaZulu-Natal, suggesting the existence of separate phylogeographic groups before their translocation into Madagascar. The estimates does not exceed the time to most recent common ancestor of *Potamochoerus* (approximately 2.71 Ma)^67^. The time of divergence between Malagasy and Zimbabwe bushpigs dramatically precedes that of any conclusive evidence of human occupation on Madagascar. Given that there is no evidence of bushpigs in Madagascar during the Holocene palaeontological or in pre-tenth-century archaeological records, the presence of bushpigs in Madagascar would have likely involved human intervention unless future fossil discoveries suggest otherwise. This early divergence indicates that the Zimbabwe population does not represent the source population of Malagasy *per-se.* Altogether, our results indicate that to identify the source population of Malagasy bushpigs, future studies should collect additional samples from locations such as Mozambique, southern Tanzania, and offshore islands—specifically the Comoros, Mafia, and Zanzibar.

The study showed that mitochondrial DNA was useful in inferring the relationships of Malagasy bushpigs but this was not the case for the nuclear DNA *GPIP* and *MC1R*. Both the phylogenetic topologies produced for *GPIP* and *MC1R* were less resolved. The *MC1R* gene was useful in discerning coat colour for breeds of domestic pigs but not in bushpigs, however further studies identifying other melanin producing genes may be suitable for similar studies^50,59,102,103^. The usage of *GPIP* was also not informative for distinguishing between bushpig populations as for domestic pig breeds which was valuable in supporting information about domestication^48,49^. Sequencing of additional mitochondrial genes could also provide a more comprehensive insight into the divergence of bushpigs from Madagascar and the mainland.

### Immunogenetic diversity of bushpigs from Madagascar

Our results show that Malagasy bushpigs have maintained similar levels of and unique genetic diversity within the important MHC class II *SLA-DQB1* ABS through diversifying selection when compared to mainland animals (Fig. 8). This suggests that founder effects, such as genetic drift and founder population size^12,28,104^, had minimal effect on the *SLA-DQB1* ABS and the adaptation to environmental challenges after the introduction to Madagascar may have driven the rise of new alleles and maintenance of genetic diversity. Most samples here are also from the tropical zones of Madagascar in contrast to the arid and temperate zones of Zimbabwe and southern Africa^105^. It is possible that the prevalence, abundance, and infection of novel parasites, driven by specific precipitation and temperature in Madagascar influenced MHC allele frequencies as has been suggested for other species^87,106,107^. In comparison, the retention of ancestral *SLA-DQB1* ABS amongst Malagasy and mainland bushpigs may indicate the necessity to combat common pathogens such as as *Taenia solium*^108,109^, *Trichinella*^110^, and *Burkloderia*^111^. However, detailed studies of bushpigs are still limited, particularly due to their elusive behaviour. Investigations into parasitic infections in both the mainland and Madagascar, along multiple habitat ranges, and pathogen-associated studies could provide in-depth insights into the roles of specific haplotypes of MHC genes^106,112,113^.

In contrast to the above, the conserved *SLA-6* haplotypes found in some of these species when observing amino acid translations (*SLA-6-*1, -4, and -7 were found in five genera: *P. larvatus*, *P. africanus*, *H. meinertzahgeni*, *S. scrofa* [wild boar], and *B. babyrussa*; and the bushpig specimen from Duisburg Zoo was identical to *S. celebensis*) are expected as this gene is often described as monomorphic^114^. *SLA-6* has a wide tissue-expression but is the least overall transcribed non-classical genes^114^ with potential roles as a membrane-anchored glycoprotein and may have roles specific to each species^115^. The lack of genetic diversity of the *SLA-6* exon-2 region, coding for the alpha-1 domain, provides little support for species-specific functions and additional sequencing of the alpha-2 domain may provide more information. There is also potential for the *SLA* non-classical genes to have similar functions to those described in humans (*HLA-E*, *HLA-F*, and *HLA-G*) such as cell population regulation (e.g. impair T cell proliferation and cytotoxicity^116^, apoptosis induction^117^), and possibly immunomodulation during pregnancy^118^.

The class III *BAG6* gene was highly conserved and indicates a retention of these haplotypes across Suidae species due to similar functions, and the low diversity found should not be due to founder effects. The Class III *BAG6* gene has been shown to play a role in the presentation of MHC class I on the cell surface and protects against cell death by metabolising defective ribosomal products^119^. The importance of this gene in African swine fever infection has been implicated in the ability of viral genes to exploit host genes to evade host immunity^60^. African swine fever is known to asymptomatically infect bushpigs but can cause high mortality rates in species of the genus *Sus*^120^. Due to the high conservation between the sequences, it is highly likely that the role of *BAG6* in ASFV resistant is small. Identical amino acid sequences in some haplotypes (*BAG6*-1, -3, -5, -6) only present in sub-Saharan African species, are distinct from those found in *Sus*. This may suggest functions that are different between genera. African swine fever infection in other species such as *B. babyrussa* and peccaries have been poorly studied and are suggested to not be affected by ASFV^121,122^. It is possible that mutations in genes can reduce activity or interfere with ASFV production^60^ and the role of *BAG6* in ASFV resistance should be studied in the context of other Suidae and Tayassuidae species as well.

## Conclusion

The current study provided genetic evidence that the bushpigs samples obtained in Madagascar are *Potamochoerus larvatus* which originated from mainland Africa and there was no genetic differentiation between eastern and western Malagasy bushpigs as suggested by previous morphological work. The introduction of bushpigs in Madagascar was associated with few maternal lineages and based on the current sampling seem closest to the sampled natural populations of Zimbabwe, but this conclusion is preliminary until more detailed phylogeographic data is available from other areas of eastern and south-eastern Africa, including southern Tanzania and Mozambique. The MHC sequences examined also showed differences between bushpigs from mainland Africa and Madagascar, with few haplotypes of the polymorphic *SLA-DQB1* shared between the two groups, suggesting they are undergoing diversification from their mainland ancestors. This also indicates that the introduction and isolation of bushpigs in Madagascar did not severely reduce their immunogenetic repertoire and appear to facilitate adaptation to new environments. Additional sequencing of specimens from other locations around Madagascar, as well as eastern Africa, is needed to provide a more in-depth indication into the source population of bushpigs introduced to Madagascar.

## Supplementary information

Supplementary Information.

## Data Availability

The CR, *cytb*, *GPIP*, *MC1R*, *SLA-DQB1*, *SLA-6*, and *BAG6* sequences generated during the current study are available in the GenBank repository under the GenBank accession numbers: MT853484–MT853538, MT864081–MT864142, MT864030–MT864080, MT864143–MT86419, MT853335–MT853419, MT853262–MT853334, and MT853420–MT853483, respectively.
