# Supplementary material for: Genetic origins and diversity of bushpigs from Madagascar (Potamochoerus larvatus, family Suidae)
Source: Sci Rep. 2020 Nov 26;10:20629. doi: 10.1038/s41598-020-77279-5 (PMC7693328; doi:10.1038/s41598-020-77279-5)
Supplement: Supplementary file 1 — Supplementary Information. [file 41598_2020_77279_MOESM1_ESM.docx]

**Genetic origins and diversity of bushpigs from Madagascar (*Potamochoerus larvatus*, family Suidae)**

Carol Lee^1^, Jenna Day^1^, Steven M Goodman^2,3^, Miguel Pedrono^4^, Guillaume Besnard^5^, Laurent Frantz^6,7^, Peter J Taylor^8,9^, Michael J Herrera^10^, Jaime Gongora^1^*

^1^Sydney School of Veterinary Science, Faculty of Science, The University of Sydney, Sydney, NSW 2006, Australia

^2^Field Museum of Natural History, Chicago, Illinois 60605, USA

^3^Association Vahatra, Antananarivo 101, Madagascar

^4^UMR ASTRE, INRAE, CIRAD, Université de Montpellier, 34398 Montpellier Cedex 5, France

^5^CNRS, UPS, IRD, Laboratoire Evolution et Diversité Biologique, UMR5174, Université Toulouse III Paul Sabatier, 31062 Toulouse, France

^6^Palaeogenomics Group, Department of Veterinary Science, Ludwig Maximilian University, Munich, Germany

^7^School of Biological and Chemical Sciences, Queen Mary University of London, London, UK

^8^School of Mathematical and Natural Sciences, University of Venda, Thohoyandou, Limpopo Province, South Africa

^9^Afromontane Research Unit and Zoology Department, University of the Free State, Qwa Qwa campus, Phuthaditjhaba 9866, South Africa

^10^Archaeological Studies Program, University of the Philippines Diliman, Quezon City 1101, Philippines

*corresponding author: [jaime.gongora@sydney.edu.au](mailto:jaime.gongora@sydney.edu.au)

| 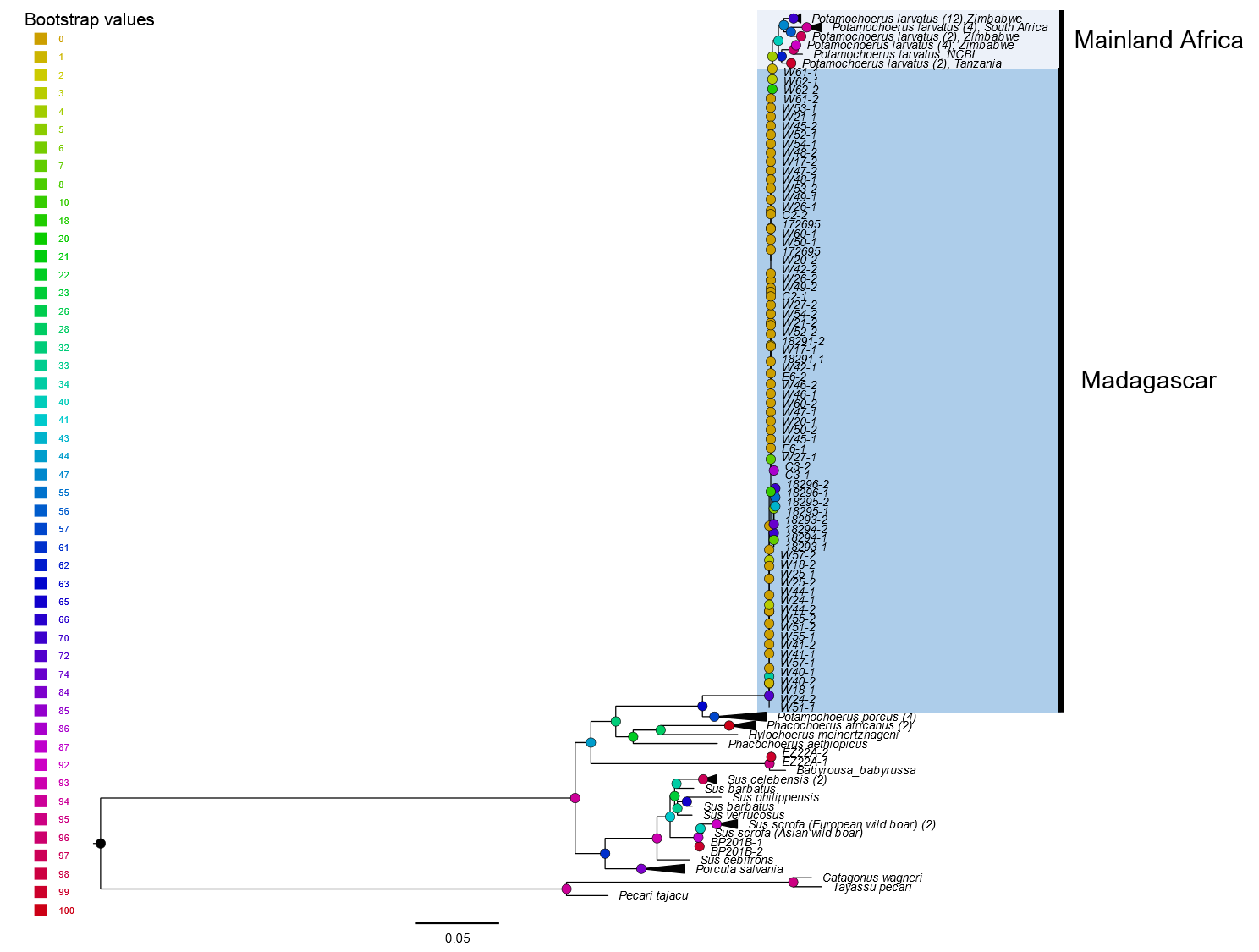 |
| --- |
| **Figure S1. Phylogenetic tree based on CR sequences indicating the position of Malagasy bushpigs as *Potamochoerus larvatus*.** A Maximum Likelihood tree was generated using the RAxML-NG v0.6.0 web-server^82^. Statistical support using a bootstrap cut-off of 0.03 (Bootstrap support indicated at each node as coloured circles). Numbers in brackets indicate the number of sequences from this study within each node. Branch scale is shown below the tree. |

| 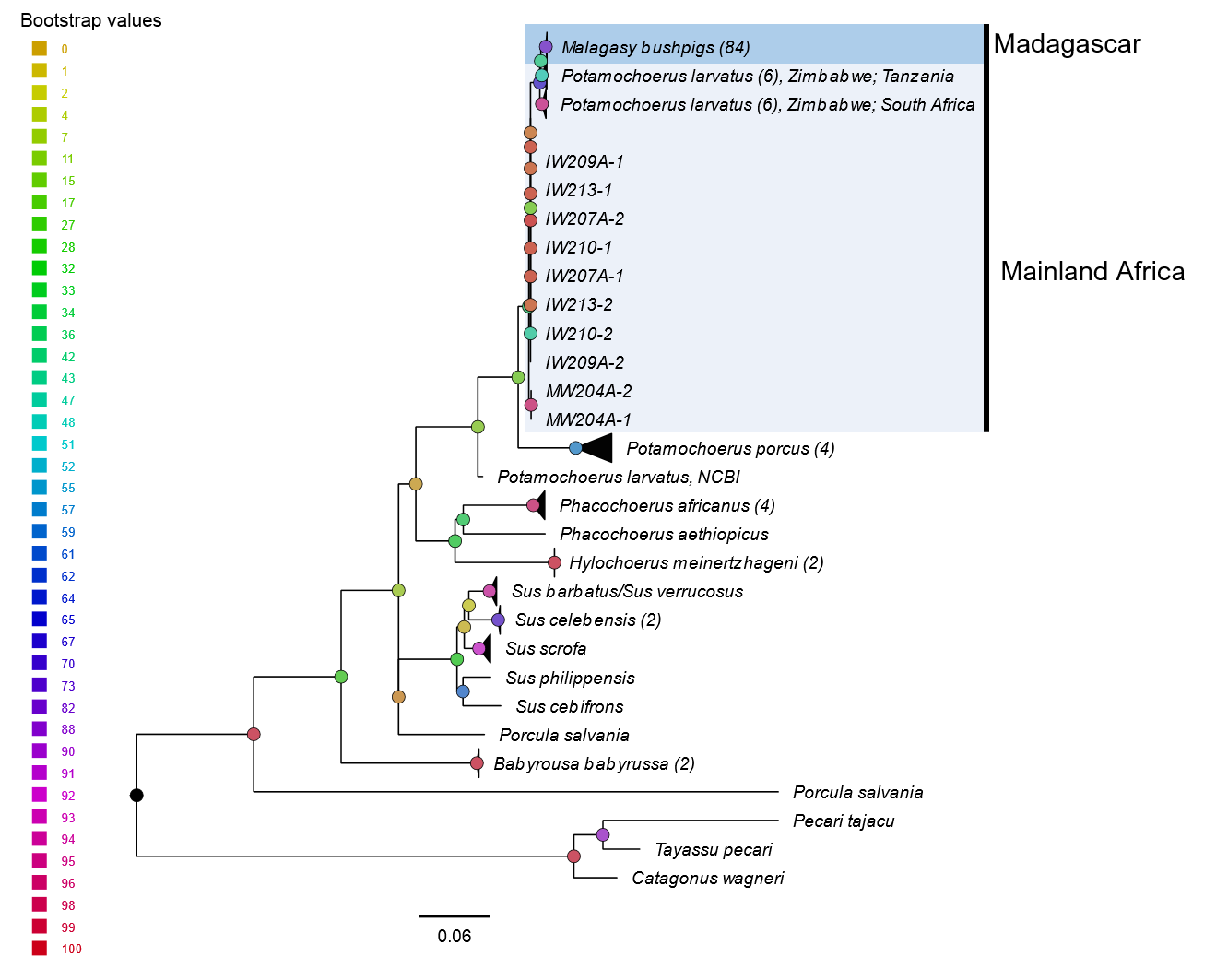 |
| --- |
| **Figure S2. Phylogenetic tree based on *cytb* sequences indicating the position of Malagasy bushpigs as *Potamochoerus larvatus*.** A Maximum Likelihood tree was generated using the RAxML-NG v0.6.0 web-server^82^. Statistical support using a bootstrap cut-off of 0.03 (Bootstrap support indicated at each node as coloured circles). Numbers in brackets indicate the number of sequences from this study within each node. Branch scale is shown below the tree. |

| 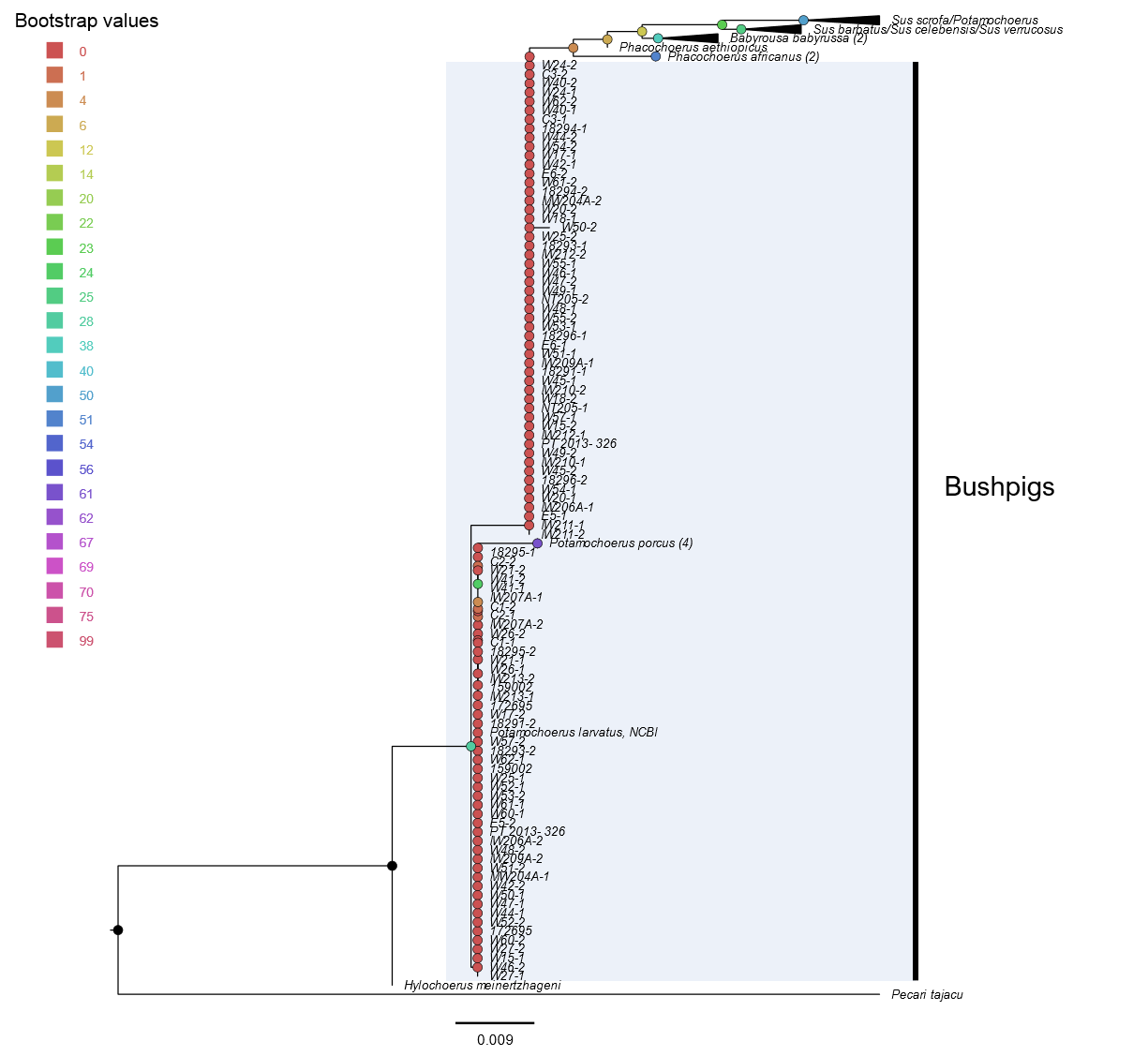 |
| --- |
| **Figure S3. Phylogenetic tree based on GPIP sequences.** A Maximum Likelihood tree was generated using the RAxML-NG v0.6.0 web-server^82^. Statistical support using a bootstrap cut-off of 0.03 (Bootstrap support indicated at each node as coloured circles). Numbers in brackets indicate the number of sequences from this study within each node. Branch scale is shown below the tree. |

| 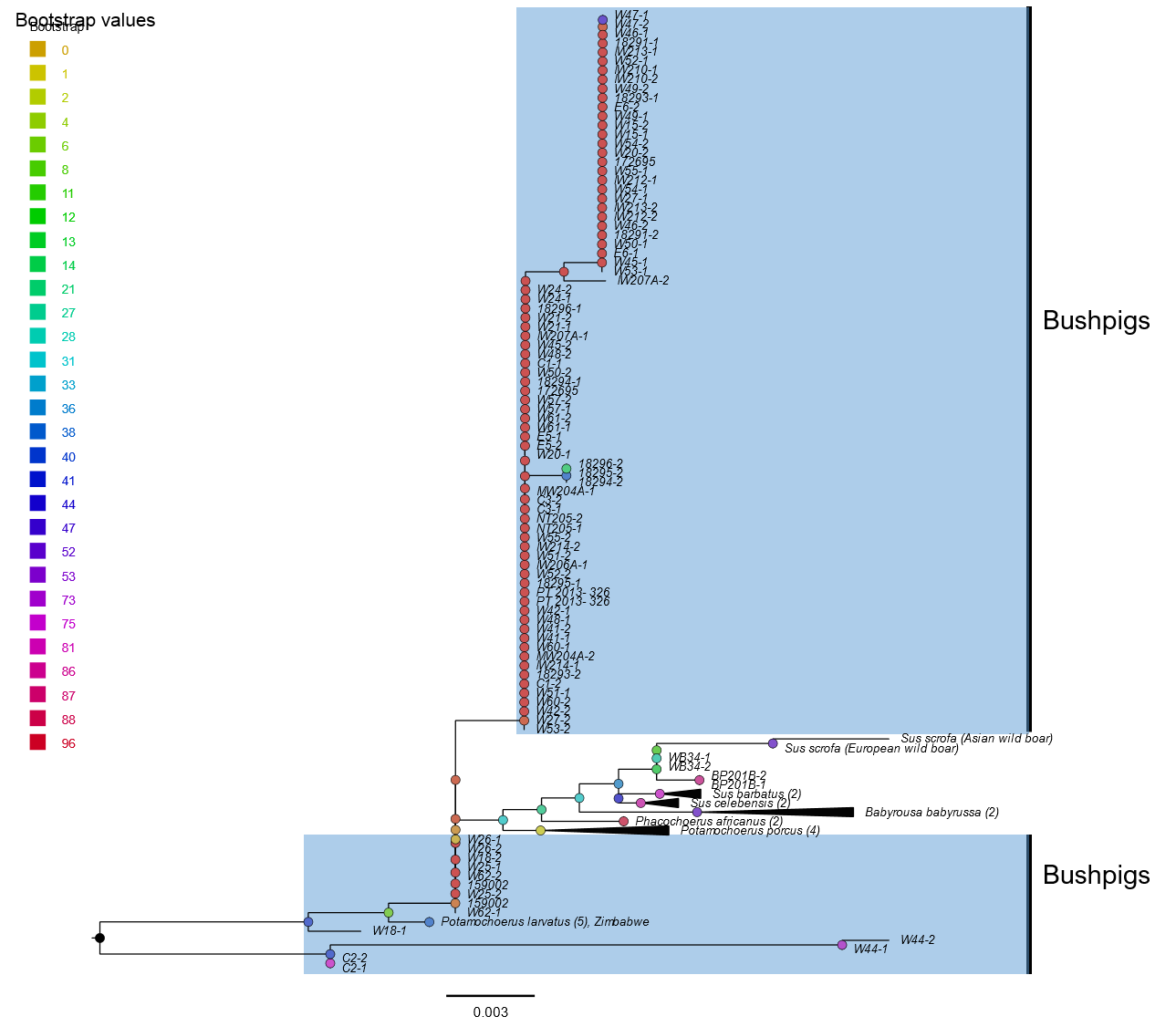 |
| --- |
| **Figure S4. Phylogenetic tree based on MC1R sequences.** A Maximum Likelihood tree was generated using the RAxML-NG v0.6.0 web-server^82^. Statistical support using a bootstrap cut-off of 0.03 (Bootstrap support indicated at each node as coloured circles). Numbers in brackets indicate the number of sequences from this study within each node. Branch scale is shown below the tree. |

| 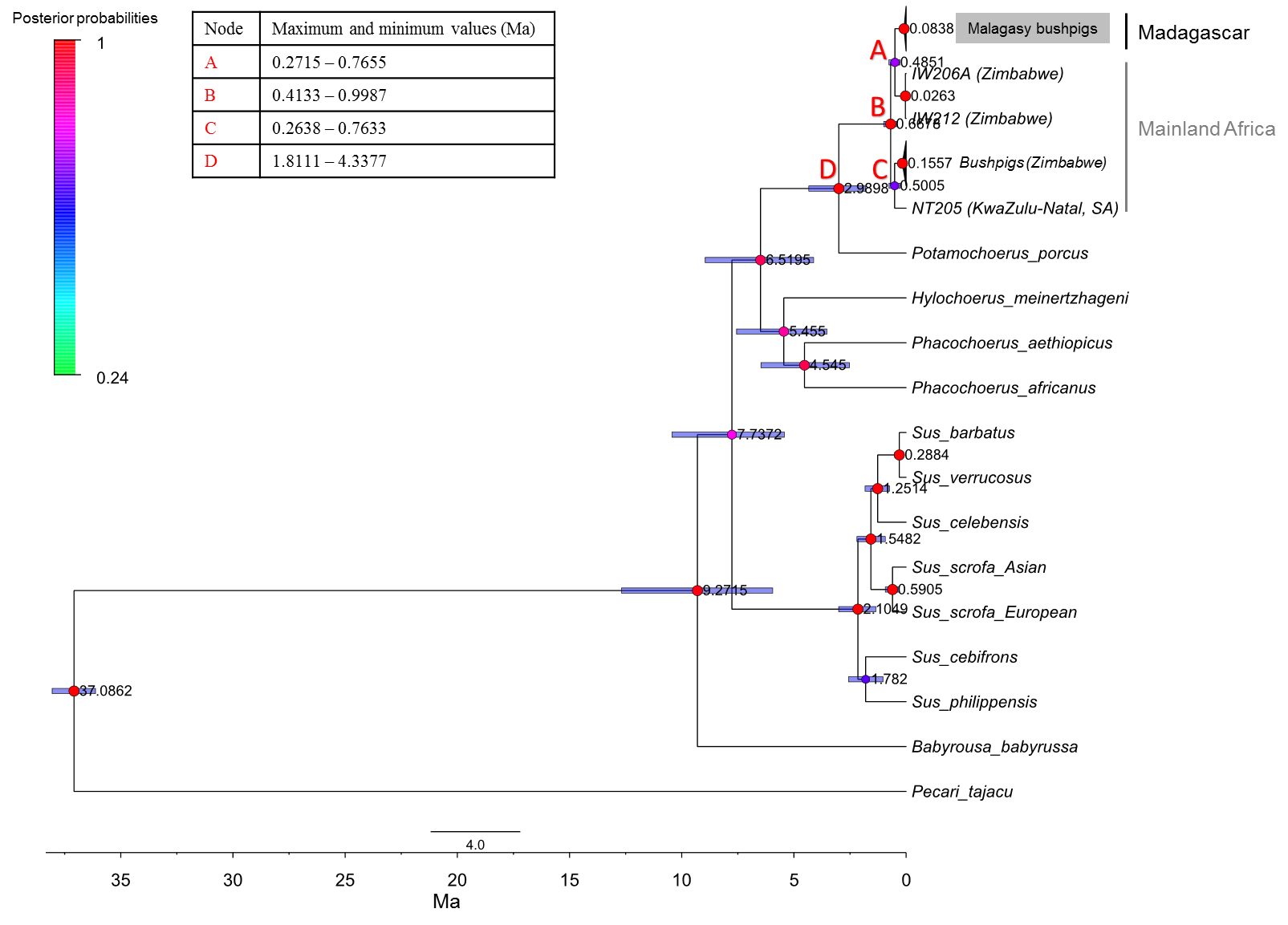 |
| --- |
| **Figure S5. Divergence time estimates from two mitochondrial regions (CR and *cytb*) of Malagasy bushpigs to extant Suidae and Tayassuidae using BEAST**^96^**.** Bayesian Posterior probabilities are indicated as coloured circles along with the median height on each node. The maximum and minimum limits of divergence time estimates are shown as node bars and the corresponding table for relevant nodes (95% Higher Posterior Density [HPD]). |

| 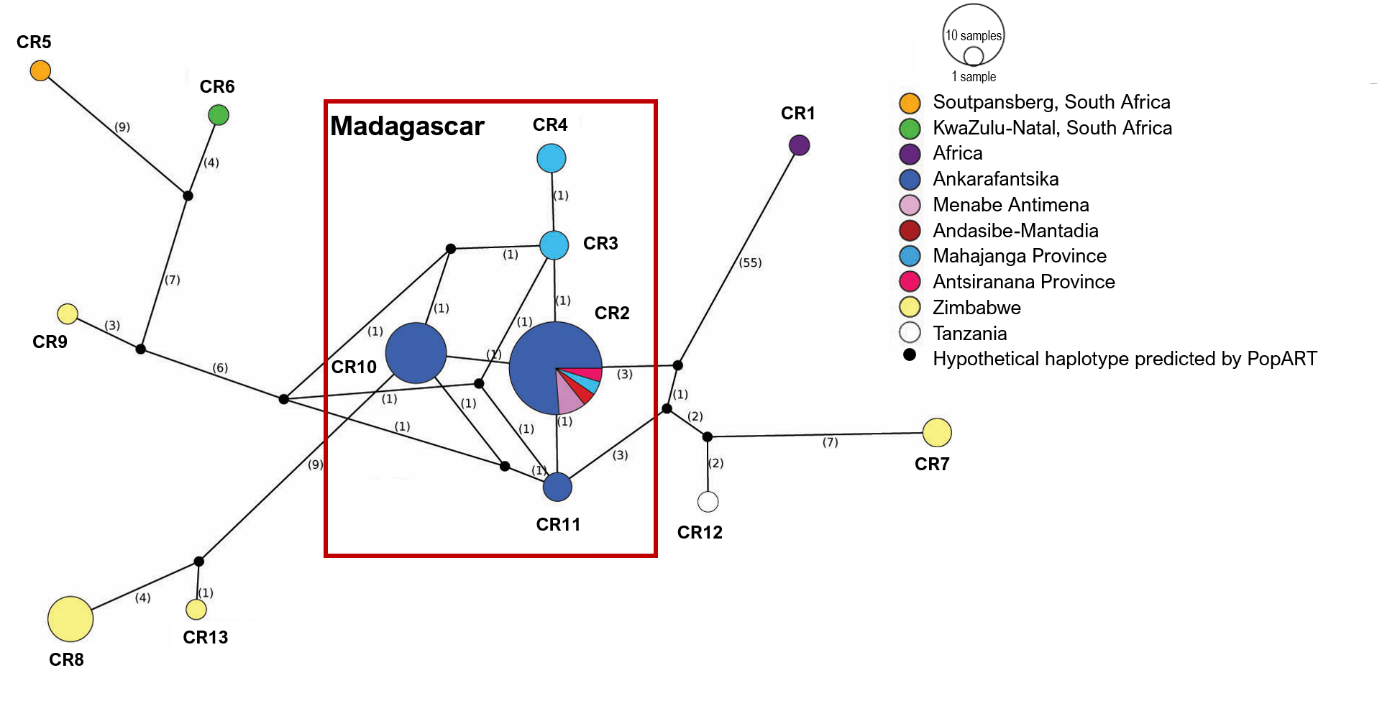 |
| --- |
| **Figure S6. Median joining network of Malagasy bushpigs based on the control region of bushpig specimens.** Coloured circles represent the haplotypes identified by DnaSP v6^68^ with each colour represented by a location as indicated by the figure key, and the size of the circle is proportional to the frequency of each haplotype. Black circles represent hypothetical haplotypes predicted by PopART^85^. Numbers in parentheses along the branches represents the mutational steps between each haplotype. |

| 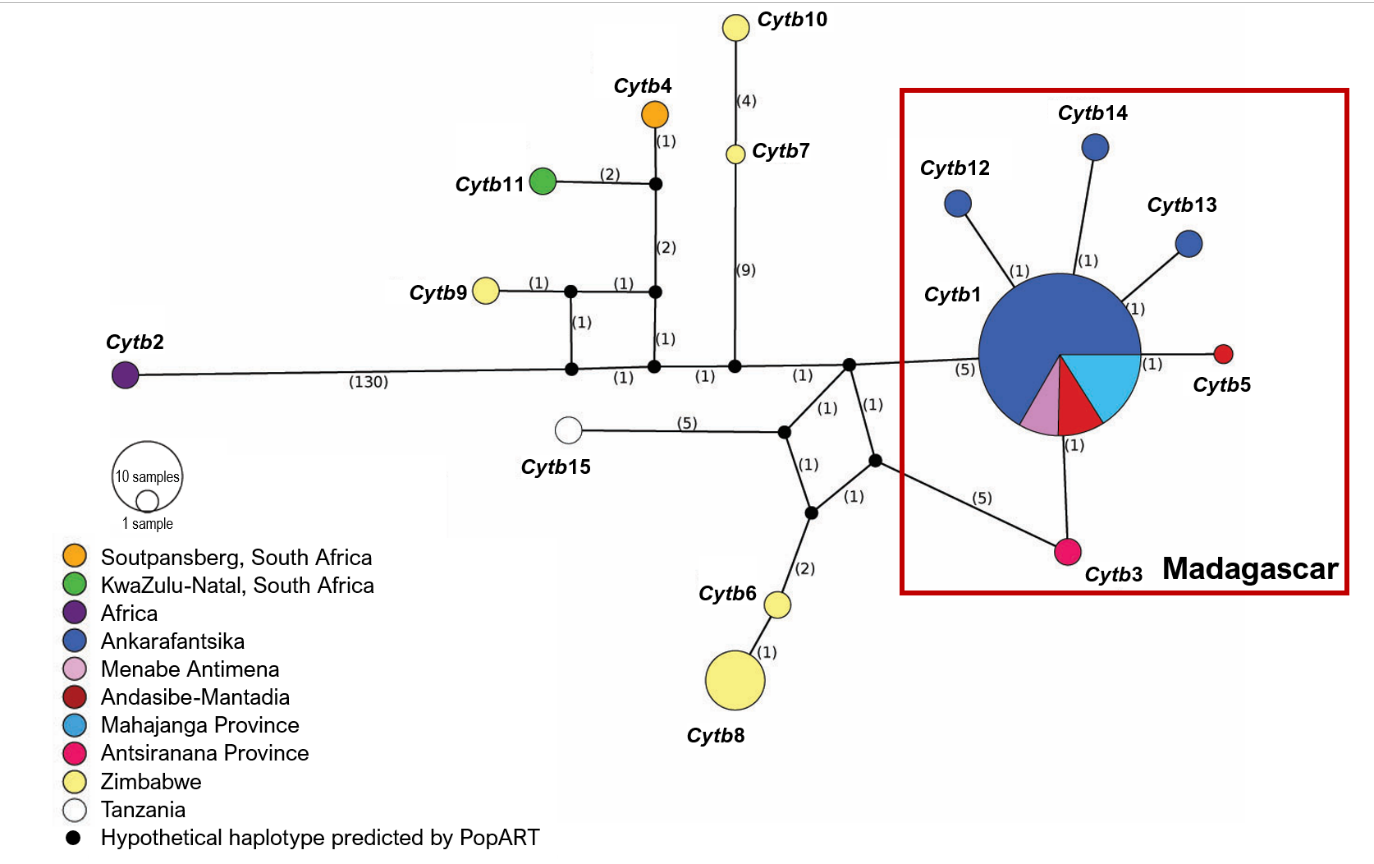 |
| --- |
| **Figure S7. Median joining network of Malagasy bushpigs based on the c*ytb* of bushpig specimens.** Coloured circles represent the haplotypes identified by DNASP v6^68^ with each colour represented by a location as indicated by the figure key, and the size of the circle is proportional to the frequency of each haplotype. Black circles represent hypothetical haplotypes predicted by PopART^85^. Numbers in parentheses along the branches represents the mutational steps between each haplotype. |

| 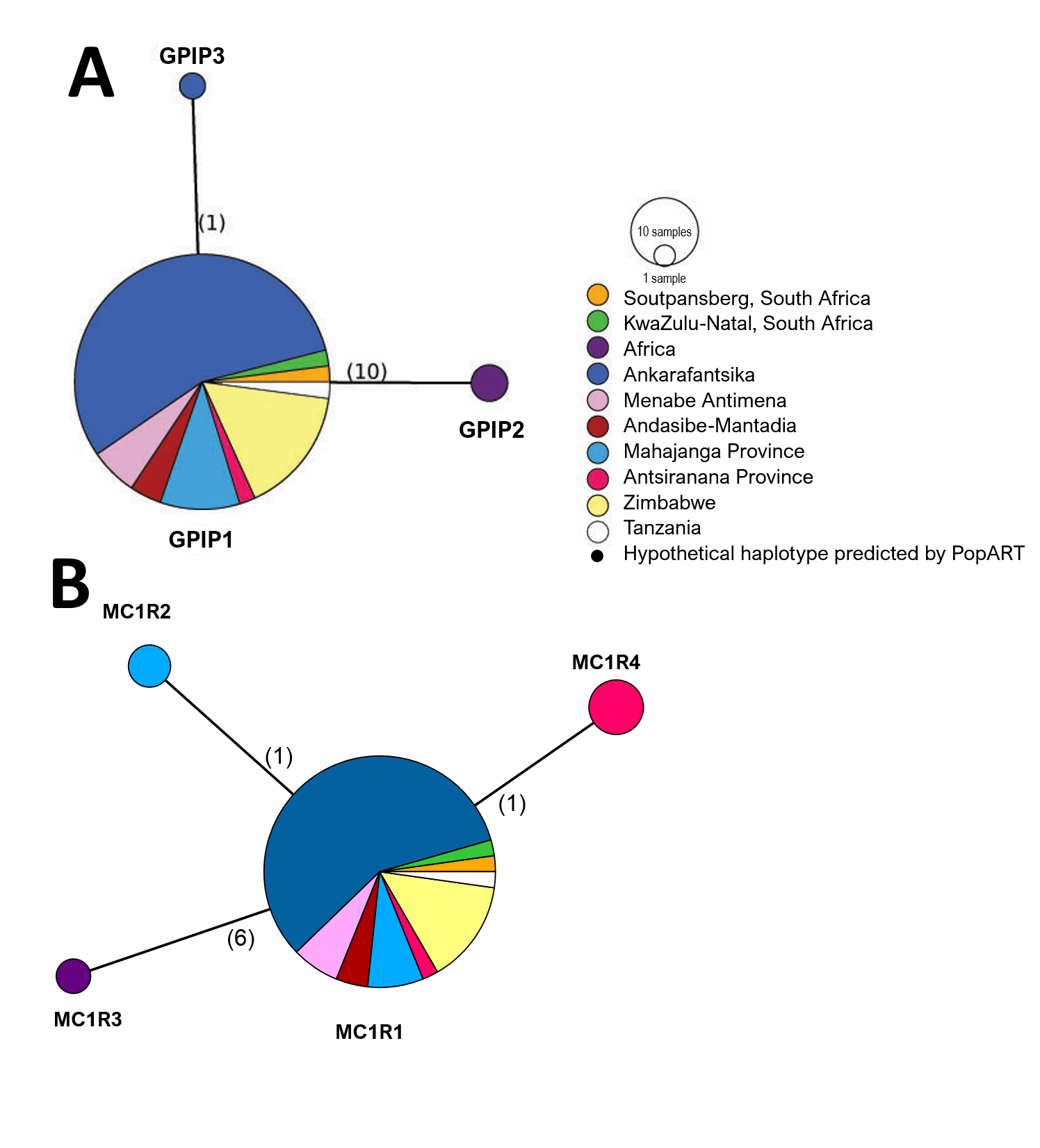 |
| --- |
| **Figure S8. Median joining network of Malagasy bushpigs based on (A) GPIP and (B) MC1R loci of bushpig specimens.** Coloured circles represent the haplotypes identified by DnaSP v6^68^ with each colour represented by a location as indicated by the figure key, and the size of the circle is proportional to the frequency of each haplotype. Black circles represent hypothetical haplotypes predicted by PopART^85^. Numbers in parentheses along the branches represents the mutational steps between each haplotype. |

| 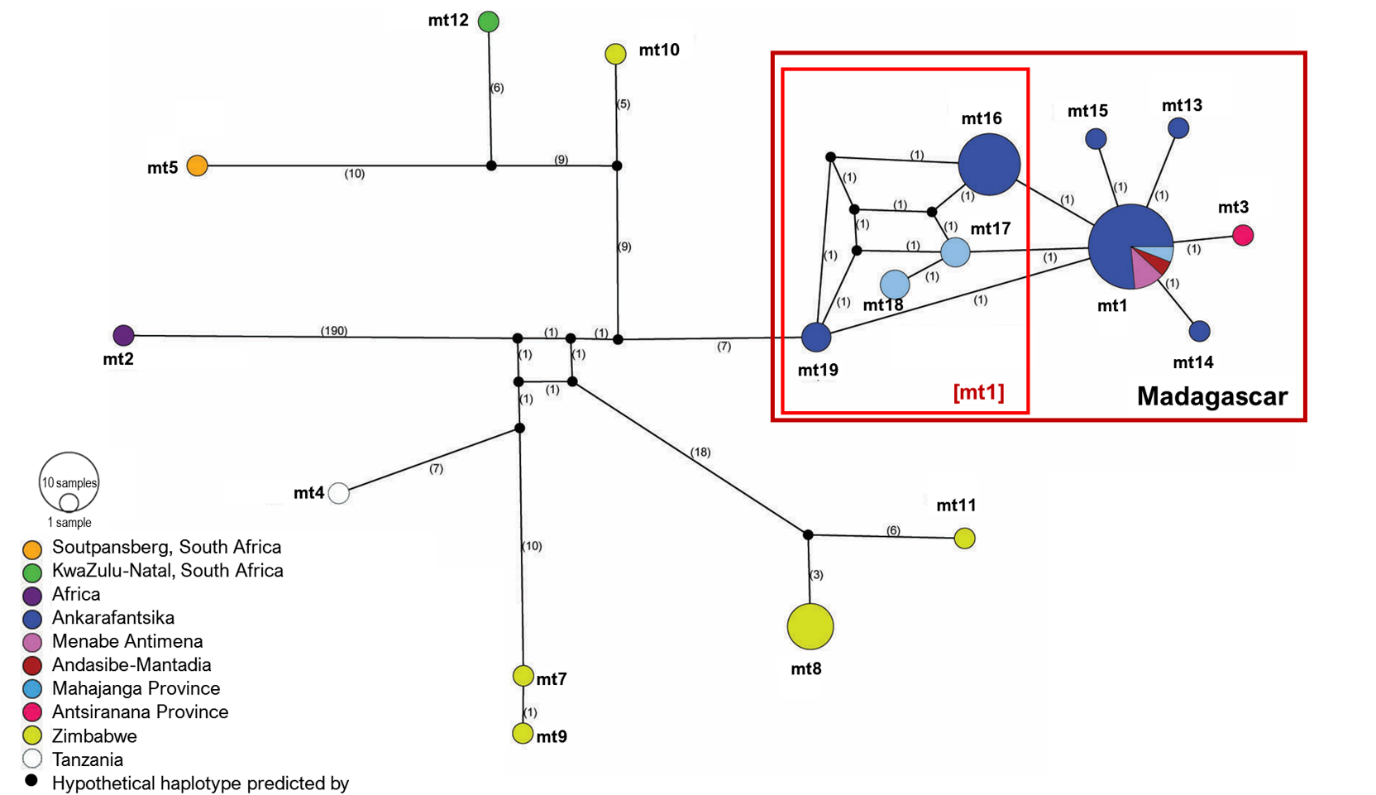 |
| --- |
| **Figure S9. Haplotype map of Malagasy and mainland bushpigs based on 98 concatenated mtDNA (CR and c*ytb*) sequences.** Coloured circles represent the haplotypes identified by DNASP v6^68^ with each colour represented by a location as indicated by the figure key, and the size of the circle is proportional to the frequency of each haplotype. Haplotypes in square parentheses indicate the haplotype number corresponding to Fig. 3 (Dataset 1b). Black circles represent hypothetical haplotypes predicted by PopART^85^. Numbers in parentheses along the branches represents the mutational steps between each haplotype. |

| 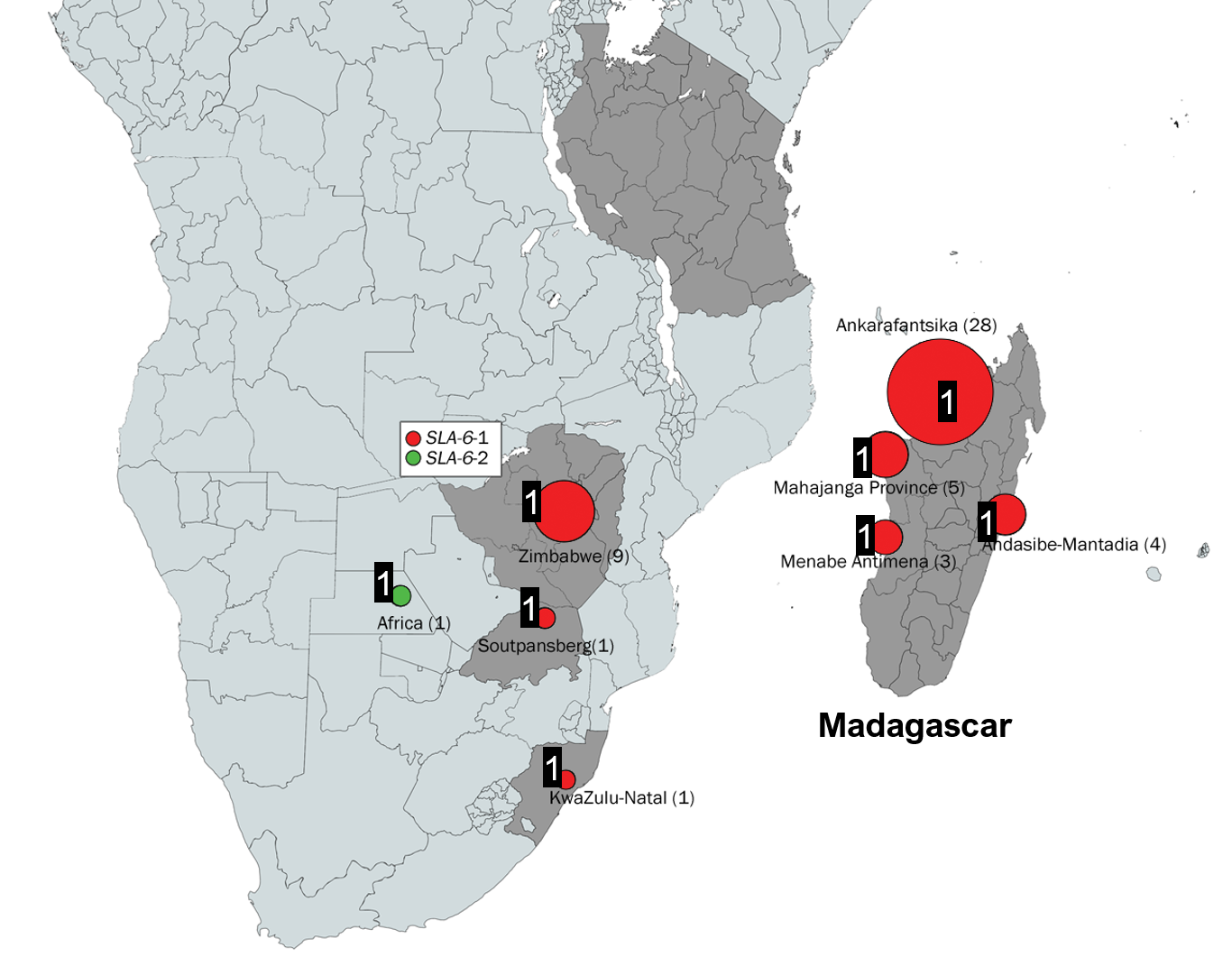 |
| --- |
| **Figure S10. Malagasy bushpig MHC class I *SLA-6* exon-2 sequence by sampling location.** Each colour indicates the different haplotypes with the number of individuals in each population shown in brackets. The number of different haplotypes within each location is highlighted by the black box adjacent to each circle. Figure includes an edited map generated by [mapchart.net](https://mapchart.net/) and haplotypes produced in PopART^85^ (<http://popart.otago.ac.nz/index.shtml>). |

| 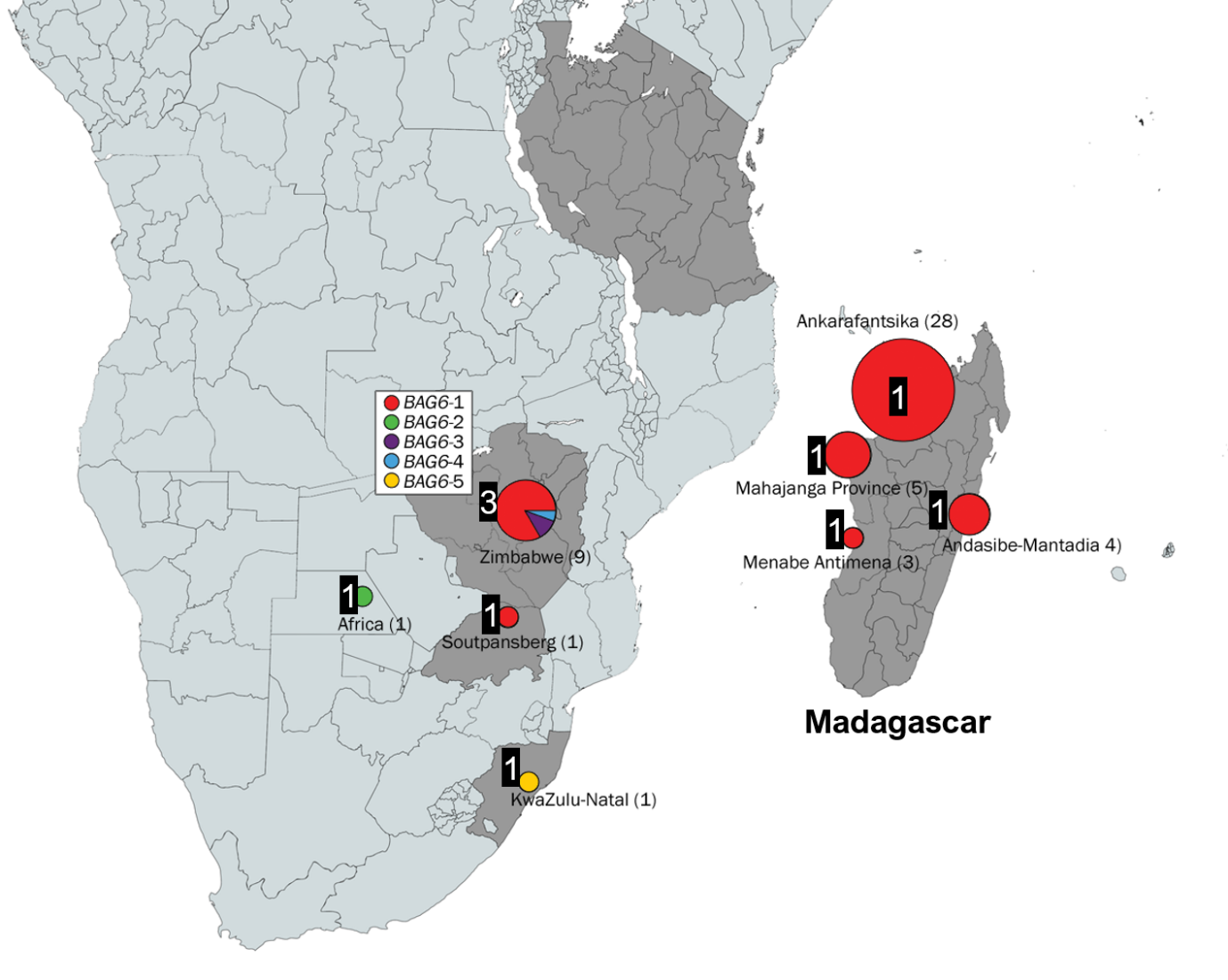 |
| --- |
| **Figure S11. Malagasy bushpig MHC class III *BAG6* loci by sampling location.** Each colour indicates the different haplotypes with the number of individuals in each population shown in brackets. The number of different haplotypes within each location is highlighted by the black box adjacent to each circle. Figure includes an edited map generated by [mapchart.net](https://mapchart.net/) and haplotypes produced in PopART^85^ (<http://popart.otago.ac.nz/index.shtml>). |

| 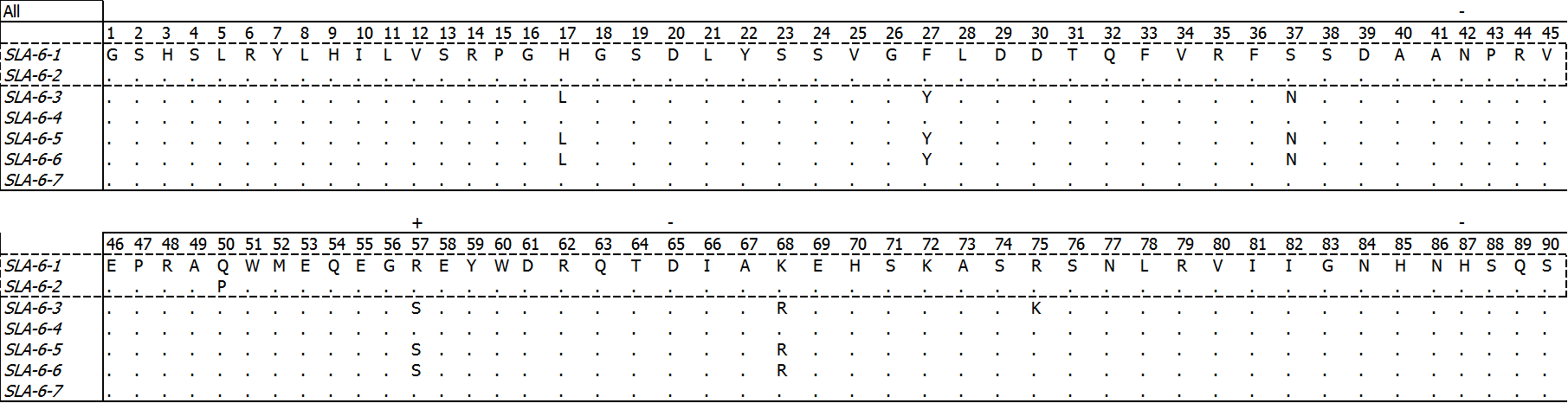 |
| --- |
| **Figure S12. Amino acid sequence and selection analysis of *SLA-6* exon 2 haplotypes.** Analysis was completed using the DataMonkey HyPhy server^86^. Dots represent identical residues to *SLA-6-*1 at the positions shown. Positions significant for positive (+) or negative (-) selection for at least two methods (MEME/FEL/SLAC/REL) are indicated. Dotted lines indicate haplotypes found in bushpigs. |

|  |
| --- |
| **Figure S13. Amino acid sequence and selection analysis for MHC class III *BAG6* (residues 450-518) haplotypes.** Analysis was completed using the DataMonkey HyPhy server^86^. Dots represent identical residues to *BAG6-*1 at the positions shown. Positions significant for positive (+) or negative (-) selection for at least two methods (MEME/FEL/SLAC/REL) are indicated. Malagasy bushpigs and mainland bushpig analysis not shown as no significant results were found. Dotted lines indicate haplotypes found in bushpigs. |

| **Table S1. Specific specimen information for this study.** Replicate samples are named ‘A’ and ‘B’ and information for sex and tissue sample were only provided if available. Museum samples are indicated and were provided by the Field Museum of Natural History, Illinois, USA. Specimens with an asterisk (*) were used in the BEAST analysis. | | | | | |
| --- | --- | --- | --- | --- | --- |
| **Sample** | **Species** | **Distribution** | **Sampling location, Longitude/Latitude (where available)** | **Sex** | **Tissue** |
| IW206A* | *Potamochoerus larvatus* | Sub-Saharan Africa | Iwaba, Zimbabwe | F | Muscle |
| IW206B | *Potamochoerus larvatus* | Sub-Saharan Africa | Iwaba, Zimbabwe | F | Muscle |
| IW207A | *Potamochoerus larvatus* | Sub-Saharan Africa | Iwaba, Zimbabwe | F | Muscle |
| IW207B | *Potamochoerus larvatus* | Sub-Saharan Africa | Iwaba, Zimbabwe | F | Muscle |
| IW209A | *Potamochoerus larvatus* | Sub-Saharan Africa | Iwaba, Zimbabwe | M | Muscle |
| IW209B | *Potamochoerus larvatus* | Sub-Saharan Africa | Iwaba, Zimbabwe | M | Muscle |
| IW210* | *Potamochoerus larvatus* | Sub-Saharan Africa | Iwaba, Zimbabwe | M | Muscle |
| IW211 | *Potamochoerus larvatus* | Sub-Saharan Africa | Iwaba, Zimbabwe | M | Muscle |
| IW212* | *Potamochoerus larvatus* | Sub-Saharan Africa | Iwaba, Zimbabwe | M | Muscle |
| IW213 | *Potamochoerus larvatus* | Sub-Saharan Africa | Iwaba, Zimbabwe | M | Muscle |
| IW214 | *Potamochoerus larvatus* | Sub-Saharan Africa | Iwaba, Zimbabwe |  |  |
| 18291A* | *Potamochoerus larvatus* | Sub-Saharan Africa | Madagascar (near NW limit of Réserve Spéciale d'Ambohijanahary, 45.2417, -18.1512, S. M. Goodman |  | Tissue |
| 18291B | *Potamochoerus larvatus* | Sub-Saharan Africa | Madagascar (near NW limit of Réserve Spéciale d'Ambohijanahary, 45.2417, -18.1512, S. M. Goodman |  | Tissue |
| 18293 | *Potamochoerus larvatus* | Sub-Saharan Africa | Madagascar (forest area about 24 km NNE Mahajanga, 27 September 2013, obtained from hunter) | F | Tissue |
| 18294* | *Potamochoerus larvatus* | Sub-Saharan Africa | Madagascar (forest area about 24 km NNE Mahajanga, 27 September 2013, obtained from hunter) | F |  |
| 18295 | *Potamochoerus larvatus* | Sub-Saharan Africa | Madagascar (forest area about 24 km NNE Mahajanga, 21 September 2013, obtained from hunter) | F |  |
| 18296 | *Potamochoerus larvatus* | Sub-Saharan Africa | Madagascar (forest area about 24 km NNE Mahajanga, 28 September 2013, obtained from hunter) | M |  |
| C1 | *Potamochoerus larvatus* | Sub-Saharan Africa | Menabe Antimena Protected Area, Madagascar |  |  |
| C2* | *Potamochoerus larvatus* | Sub-Saharan Africa | Menabe Antimena Protected Area, Madagascar |  |  |
| C3 | *Potamochoerus larvatus* | Sub-Saharan Africa | Menabe Antimena Protected Area, Madagascar |  |  |
| E3 | *Potamochoerus larvatus* | Sub-Saharan Africa | (west of the) Mantadia National Park, Madagascar |  |  |
| E4 | *Potamochoerus larvatus* | Sub-Saharan Africa | (west of the) Mantadia National Park, Madagascar |  |  |
| E5 | *Potamochoerus larvatus* | Sub-Saharan Africa | (west of the) Mantadia National Park, Madagascar |  |  |
| E6* | *Potamochoerus larvatus* | Sub-Saharan Africa | (west of the) Mantadia National Park, Madagascar |  |  |
| W15 | *Potamochoerus larvatus* | Sub-Saharan Africa | (north of the) Ankarafantsika National Park, Madagascar |  |  |
| W17 | *Potamochoerus larvatus* | Sub-Saharan Africa | (north of the) Ankarafantsika National Park, Madagascar |  |  |

| W18 | *Potamochoerus larvatus* | Sub-Saharan Africa | (north of the) Ankarafantsika National Park, Madagascar | | | |  |  |
| --- | --- | --- | --- | --- | --- | --- | --- | --- |
| W20 | *Potamochoerus larvatus* | Sub-Saharan Africa | (north of the) Ankarafantsika National Park, Madagascar | | | |  |  |
| W21 | *Potamochoerus larvatus* | Sub-Saharan Africa | (north of the) Ankarafantsika National Park, Madagascar | | | |  |  |
| W24* | *Potamochoerus larvatus* | Sub-Saharan Africa | (north of the) Ankarafantsika National Park, Madagascar | | | |  |  |
| W25 | *Potamochoerus larvatus* | Sub-Saharan Africa | (north of the) Ankarafantsika National Park, Madagascar | | | |  |  |
| W26 | *Potamochoerus larvatus* | Sub-Saharan Africa | (north of the) Ankarafantsika National Park, Madagascar | | | |  |  |
| W27 | *Potamochoerus larvatus* | Sub-Saharan Africa | (north of the) Ankarafantsika National Park, Madagascar | | | |  |  |
| W40 | *Potamochoerus larvatus* | Sub-Saharan Africa | (north of the) Ankarafantsika National Park, Madagascar | | | |  |  |
| W41 | *Potamochoerus larvatus* | Sub-Saharan Africa | (north of the) Ankarafantsika National Park, Madagascar | | | |  |  |
| W42 | *Potamochoerus larvatus* | Sub-Saharan Africa | (north of the) Ankarafantsika National Park, Madagascar | | | |  |  |
| W44 | *Potamochoerus larvatus* | Sub-Saharan Africa | (north of the) Ankarafantsika National Park, Madagascar | | | |  |  |
| W45 | *Potamochoerus larvatus* | Sub-Saharan Africa | (north of the) Ankarafantsika National Park, Madagascar | | | |  |  |
| W46 | *Potamochoerus larvatus* | Sub-Saharan Africa | (north of the) Ankarafantsika National Park, Madagascar | | | |  |  |
| W47 | *Potamochoerus larvatus* | Sub-Saharan Africa | (north of the) Ankarafantsika National Park, Madagascar | | | |  |  |
| W48 | *Potamochoerus larvatus* | Sub-Saharan Africa | (north of the) Ankarafantsika National Park, Madagascar | | | |  |  |
| W49 | *Potamochoerus larvatus* | Sub-Saharan Africa | (north of the) Ankarafantsika National Park, Madagascar | | | |  |  |
| W50 | *Potamochoerus larvatus* | Sub-Saharan Africa | (north of the) Ankarafantsika National Park, Madagascar | | | |  |  |
| W51 | *Potamochoerus larvatus* | Sub-Saharan Africa | (north of the) Ankarafantsika National Park, Madagascar | | | |  |  |
| W52 | *Potamochoerus larvatus* | Sub-Saharan Africa | (north of the) Ankarafantsika National Park, Madagascar | | | |  |  |
| W53 | *Potamochoerus larvatus* | Sub-Saharan Africa | (north of the) Ankarafantsika National Park, Madagascar | | | |  |  |
| W54 | *Potamochoerus larvatus* | Sub-Saharan Africa | (north of the) Ankarafantsika National Park, Madagascar | | | |  |  |
| W55* | *Potamochoerus larvatus* | Sub-Saharan Africa | (north of the) Ankarafantsika National Park, Madagascar | | | |  |  |
| W57 | *Potamochoerus larvatus* | Sub-Saharan Africa | (north of the) Ankarafantsika National Park, Madagascar | | | |  |  |
| W60 | *Potamochoerus larvatus* | Sub-Saharan Africa | (north of the) Ankarafantsika National Park, Madagascar | | | |  |  |
| W61 | *Potamochoerus larvatus* | Sub-Saharan Africa | (north of the) Ankarafantsika National Park, Madagascar | | | |  |  |
| W62* | *Potamochoerus larvatus* | Sub-Saharan Africa | (north of the) Ankarafantsika National Park, Madagascar | | | |  |  |
| NT205* | *Potamochoerus larvatus* | Sub-Saharan Africa | KwaZulu-Natal, South Africa | | | | M | Muscle |
| PT 2013-326A | *Potamochoerus larvatus* | Sub-Saharan Africa | Soutpansberg, Limpopo Province, South Africa | | | |  | Skin |
| PT 2013-326B | *Potamochoerus larvatus* | Sub-Saharan Africa | Soutpansberg, Limpopo Province, South Africa | | | |  | Skin |
| BP201B | *Potamochoerus larvatus* | Sub-Saharan Africa | Tervuren, Belgium | | | | Foetus | Heart |
| MW204A* | *Potamochoerus larvatus* | Sub-Saharan Africa | Zimbabwe | | | | M | Blood |
| MW204B | *Potamochoerus larvatus* | Sub-Saharan Africa | Zimbabwe | | | | M | Blood |
| VL208A | *Potamochoerus porcus* | Sub-Saharan Africa | Duisberg Zoo, Germany | | | | M | Muscle |
| RZ203 | *Potamochoerus porcus* | Sub-Saharan Africa | Rotterdam Zoo, Netherlands | | | | Neonate | Blood |
| RH200 | *Potamochoerus porcus* | Sub-Saharan Africa | Tervuren, Belgium | | | | M | Heart |
| IW108A | *Phacochoerus africanus* | Sub-Saharan Africa | Iwaba, Zimbabwe | | | | M | Muscle |
| FH2 | *Hylochoerus meinertzhageni* | Sub-Saharan Africa | Uganda | | | | M | Liver |
| WB34 | *Sus scrofa* | Europe | Yorkshire Farm, UK | | | | M | Blood |
| LZ3792 | *Sus barbatus* | Southeast Asia | ZSL Animal Hospital, UK | | | | F | Blood |
| SU332A | *Sus celebensis* | Southeast Asia | Sulawesi mainland, Indonesia | | | | M | Muscle |
| EZ22A | *Babyrousa babyrussa* | Southeast Asia | Edinburgh Zoo, UK | | | | M | Blood |
| 90 | *Pecari tajacu* | North, Central and South America | Barranquilla, Colombia | | | | F | Blood |
| 93 | *Pecari tajacu* | North, Central and South America | Barranquilla, Colombia | | | | F | Blood |
| 98 | *Pecari tajacu* | North, Central and South America | Barranquilla, Colombia | | | | F | Blood |
| 99 | *Pecari tajacu* | North, Central and South America | Barranquilla, Colombia | | | | F | Blood |
| 105 | *Pecari tajacu* | North, Central and South America | Barranquilla, Colombia | | | | M | Blood |
| 106 | *Pecari tajacu* | North, Central and South America | Barranquilla, Colombia | | | | M | Blood |
| 108 | *Pecari tajacu* | North, Central and South America | Barranquilla, Colombia | | | | M | Blood |
| 110 | *Pecari tajacu* | North, Central and South America | Barranquilla, Colombia | | | | M | Blood |
| 112 | *Pecari tajacu* | North, Central and South America | Barranquilla, Colombia | | | | M | Blood |
| 114 | *Pecari tajacu* | North, Central and South America | Barranquilla, Colombia | | | | M | Blood |
| 60 | *Pecari tajacu* | North, Central and South America | Macagual, Colombia | | | | M | Blood |
| 62 | *Pecari tajacu* | North, Central and South America | Macagual, Colombia | | | | F | Blood |
| 63 | *Pecari tajacu* | North, Central and South America | Macagual, Colombia | | | | M | Blood |
| 64 | *Pecari tajacu* | North, Central and South America | Macagual, Colombia | | | | M | Blood |
| 65 | *Pecari tajacu* | North, Central and South America | Macagual, Colombia | | | | M | Blood |
| 66 | *Pecari tajacu* | North, Central and South America | Macagual, Colombia | | | | M | Blood |
| 67 | *Pecari tajacu* | North, Central and South America | Macagual, Colombia | | | | M | Blood |
| 68 | *Pecari tajacu* | North, Central and South America | Macagual, Colombia | | | | F | Blood |
| 69 | *Pecari tajacu* | North, Central and South America | Macagual, Colombia | | | | F | Blood |
| 70 | *Pecari tajacu* | North, Central and South America | Macagual, Colombia | | | | M | Blood |
| **Museum specimens** | | | | | | | | |
| **Catalogue number** |  |  |  | **Longitude** | **Latitude** | **Date of collection** |  |  |
| FMNH 172695 | *Potamochoerus larvatus* | Sub-Saharan Africa | *Forêt d*' *Antsahabe*, south of Daraina, Antsiranana Province, Madagascar | 49.6167 | -13.255 | 5 Nov 2002 |  | Muscle |
| FMNH 175736 | *Potamochoerus larvatus* | Sub-Saharan Africa | Vilanandro, Mahajanga Province, Madagascar |  |  | 10 Oct 2002 | F | Skull |
| FMNH 159002 | *Potamochoerus larvatus* | Sub-Saharan Africa | Kwamgumi Forest Reserve, Tanzania (Tanga Region) | 38.73 | -4.9417 | 18 July 1994 | M | Skull |

| **Table S2. Details of NCBI sequences used for phylogenetic analysis.** The species alignment name and concatenated sequences (NCBI accession numbers and region) are provided as a row. N/A represent sequences that were not available for that species loci. | | | | | |
| --- | --- | --- | --- | --- | --- |
|  | **Control Region** | ***Cytb*** | **GPIP** | **MC1R** | **References** |
| **Species** | **Accession no.** | **Accession no.** | **Accession no.** | **Accession no.** |  |
| *Sus scrofa* (Asian wild boar) | EF545579.1:1-1254 | EF545579.1:15419-16558 | AF181958.1 | AY365254.1 | 47, 75 |
| *Sus scrofa* (European wild boar) | AB015095.1 | AB015083.1 | AY629190.1 | AM492524.1 | 68,77 |
| *Sus cebifrons* | KF952600.1:1-1044 | KF952600.1:15202-16341 | N/A | N/A |  |
| *Sus celebensis* | KM203891.1:1-1041 | KM203891.1:15209-16348 | GQ338973.1 | N/A | 69,77 |
| *Sus barbatus* 1 | DQ779476.1 | AM492662.1 | GQ338971.1 | N/A | 69–71 |
| *Sus barbatus* 2 | DQ779457.1 | AY534297.1 | N/A | N/A | 70,72 |
| *Sus verrucosus* | KF926379.1:1-1047 | KF926379.1:15207-16346 | GQ338972.1 | N/A |  |
| *Sus philippensis* | DQ779369 | AY920905 | N/A | N/A |  |
| *Porcula salvania* 1 | EU107789.1:243-1580 | EU107789.1:1-110 | N/A | N/A |  |
| *Porcula salvania* 2 | EF472247.1 | EU107788.1 | N/A | N/A |  |
| *Potamochoerus porcus* 1 | JN632688.1:15440-16693 | JN632688.1:14168-15307 | FJ665498.1 | FJ665487.1 | 74,77 |
| *Potamochoerus porcus* 2 | NC_020737.1:15440-16693 | NC_020737.1:14168-15307 | N/A | N/A | 77 |
| *Babyrousa babyrussa* | GQ338961.1 | Z50106.1 | GQ338980.1 | FJ773357.1 | 69,71,72,77 |
| *Hylochoerus meinertzhageni* | GQ338959.1 | GQ338968.1 | GQ338977.1 | N/A | 69 |
| *Potamochoerus larvatus* | GQ338957.1 | GQ338966.1 | GQ338975.1 | N/A | 69 |
| *Phacochoerus africanus* 1 | DQ409327.1:1-1276 | DQ409327.1:15447-16586 | GQ338978.1 | N/A | 69,75 |
| *Phacochoerus africanus* 2 | NC_008830.1:1-1276 | NC_008830.1:15447-16586 | N/A | N/A | 75 |
| *Phacochoerus aethiopicus* | AJ314535.1 | AJ314549.1 | GQ338979.1 | N/A | 69 |
| *Tayasu pecari* | AY546516.1 | AY726775.1 | N/A | N/A | 75 |
| *Pecari tajacu* | JN632682.1:15434-16827 | JN632682.1:14160-15299 | AY568037.1 | N/A | 75, 77 |
| *Catagonus wagneri* | AY546521.1 | U66291.1 | N/A | N/A | 75 |

| **Table S3. Partitions and nucleotide substation models used for BEAST analysis.** | |
| --- | --- |
| Best Nucleotide Model | Partition |
| HKY+G | CR |
| HKY+G | CYTB_1 |
| HKY+I | CYTB_2 |
| HKY+G | CYTB_3 |

| **Table S4. Summary of *SLA-DQB1* exon-2 haplotypes found in each species and location.** Sampling numbers and haplotype frequencies (%) are based on number of individuals passing quality filtering. Frequencies are provided for each species and location.Suidae and Tayassuidae species are as follows: *Pola*: *P. larvatus, Popo: P. porcus, Phaf: P. africanus, Hyme: H. meinertzhageni, Baba: Babyrousa babyrussa, Susc: S. scrofa, Suba: S. barbatus, Suce: S. celebensis, Peta: P. tajacu.*). Haplotypes whose amino acid translation are identical are shown and labelled ‘AA’ followed by a number. | | | | | | | | | | | | | | | | | | | | | | | | | | | | | | | | | | | | | | | |
| --- | --- | --- | --- | --- | --- | --- | --- | --- | --- | --- | --- | --- | --- | --- | --- | --- | --- | --- | --- | --- | --- | --- | --- | --- | --- | --- | --- | --- | --- | --- | --- | --- | --- | --- | --- | --- | --- | --- | --- |
| **Identical amino acid** |  |  |  |  |  |  |  |  |  | **AA1** |  |  |  |  |  |  |  |  |  |  |  |  |  |  |  |  |  |  | **AA1** |  |  |  |  |  |  |  |  |  |  |
| **Location/species** | ***N*** | ***DQB1-*1** | ***DQB1-*2** | ***DQB1-*3** | ***DQB1-*4** | ***DQB1-*5** | ***DQB1-*6** | ***DQB1-*7** | ***DQB1-*8** | ***DQB1-*9** | ***DQB1-*10** | ***DQB1-*11** | ***DQB1-*12** | ***DQB1-*13** | ***DQB1-*14** | ***DQB1-*15** | ***DQB1-*16** | ***DQB1-*17** | ***DQB1-*18** | ***DQB1-*19** | ***DQB1-*20** | ***DQB1-*21** | ***DQB1-*22** | ***DQB1-*23** | ***DQB1-*24** | ***DQB1-*25** | ***DQB1-*26** | ***DQB1-*27** | ***DQB1-*28** | ***DQB1-*29** | ***DQB1-*30** | ***DQB1-*31** | ***DQB1-*32** | ***DQB1-*33** | ***DQB1-*34** | ***DQB1-*35** | ***DQB1-*36** | ***DQB1-*37** | ***DQB1-*38** |
| **Bushpigs (*Pola*)** | 47 | 9.1 | 2.3 | 2.3 | 11.4 | 2.3 | 20.5 | 2.3 | 31.8 | 15.9 | 2.3 | 2.3 | 2.3 | 2.3 | 2.3 | 6.8 | 2.3 | 2.3 | 2.3 | 20.5 | 4.5 | 2.3 | 2.3 | 2.3 | 2.3 | 2.3 | 2.3 | 2.3 | 2.3 |  |  |  |  |  |  |  |  |  |  |
| **Madagascar** | 35 |  |  |  | 14.3 | 2.9 | 25.7 | 2.9 | 37.1 | 11.4 | 2.9 | 2.9 | 2.9 |  |  |  |  |  |  | 25.7 | 5.7 | 2.9 | 2.9 | 2.9 | 2.9 | 2.9 | 2.9 | 2.9 | 2.9 |  |  |  |  |  |  |  |  |  |  |
| Ankarafantsika | 28 |  |  |  | 10.7 |  | 21.4 |  | 42.9 | 14.3 |  |  |  |  |  |  |  |  |  | 32.1 | 7.1 | 3.6 | 3.6 | 3.6 | 3.6 | 3.6 | 3.6 | 3.6 | 3.6 |  |  |  |  |  |  |  |  |  |  |
| Mahajanga Province (general) | - |  |  |  |  |  |  |  |  |  |  |  |  |  |  |  |  |  |  |  |  |  |  |  |  |  |  |  |  |  |  |  |  |  |  |  |  |  |  |
| Menabe Antimena | 3 |  |  |  | 66.7 | 33.3 | 33.3 | 33.3 |  |  |  |  |  |  |  |  |  |  |  |  |  |  |  |  |  |  |  |  |  |  |  |  |  |  |  |  |  |  |  |
| East Madagascar  (Andasibe-Mantadia) | 4 |  |  |  |  |  | 50 |  | 25 |  | 25 | 25 | 25 |  |  |  |  |  |  |  |  |  |  |  |  |  |  |  |  |  |  |  |  |  |  |  |  |  |  |
| “North” Madagascar  (Antsiranana Province) | - |  |  |  |  |  |  |  |  |  |  |  |  |  |  |  |  |  |  |  |  |  |  |  |  |  |  |  |  |  |  |  |  |  |  |  |  |  |  |
| **Mainland Africa** | 12 | 35.3 | 5.9 | 5.9 |  |  |  |  | 5.9 | 17.6 |  |  |  | 11.8 | 11.8 | 23.5 | 11.8 | 11.8 | 5.9 |  |  |  |  |  |  |  |  |  |  |  |  |  |  |  |  |  |  |  |  |
| Zimbabwe | 9 | 44.4 |  |  |  |  |  |  |  | 22.2 |  |  |  | 11.1 | 11.1 | 33.3 | 11.1 | 11.1 |  |  |  |  |  |  |  |  |  |  |  |  |  |  |  |  |  |  |  |  |  |
| KwaZulu-Natal, SA | 1 |  |  |  |  |  |  |  |  |  |  |  |  |  |  |  |  |  | 100 |  |  |  |  |  |  |  |  |  |  |  |  |  |  |  |  |  |  |  |  |
| Soutpansberg, SA | 1 |  |  |  |  |  |  |  | 100 | 100 |  |  |  |  |  |  |  |  |  |  |  |  |  |  |  |  |  |  |  |  |  |  |  |  |  |  |  |  |  |
| Tanzania | - |  |  |  |  |  |  |  |  |  |  |  |  |  |  |  |  |  |  |  |  |  |  |  |  |  |  |  |  |  |  |  |  |  |  |  |  |  |  |
| Africa | 1 |  | 100 | 100 |  |  |  |  |  |  |  |  |  |  |  |  |  |  |  |  |  |  |  |  |  |  |  |  |  |  |  |  |  |  |  |  |  |  |  |
| **Other species** |  |  |  |  |  |  |  |  |  |  |  |  |  |  |  |  |  |  |  |  |  |  |  |  |  |  |  |  |  |  |  |  |  |  |  |  |  |  |  |
| *Popo* | 3 |  |  |  |  |  |  |  |  |  |  |  |  |  |  | 33.3 |  |  |  |  |  |  |  |  |  |  |  |  |  |  |  |  |  | 66.7 |  |  |  |  |  |
| *Phaf* | 1 |  |  |  |  |  |  |  |  |  |  |  |  |  |  |  |  |  |  |  |  |  |  |  |  |  |  |  |  |  |  |  |  |  |  |  | 100 |  |  |
| *Hyme* | 1 |  |  |  |  |  |  |  |  |  |  |  |  |  |  |  |  |  |  |  |  |  |  |  |  |  |  |  |  |  |  | 100 | 100 |  |  |  |  |  |  |
| *Baba* | 1 |  |  |  |  |  |  |  |  |  |  |  |  |  |  |  |  |  |  |  |  |  |  |  |  |  |  |  |  |  |  |  |  |  |  |  |  | 100 | 100 |
| *Susc* | 1 |  |  |  |  |  |  |  |  |  |  |  |  |  |  |  |  |  |  |  |  |  |  |  |  |  |  |  |  |  |  |  |  |  | 100 |  |  |  |  |
| *Suba* | 1 |  |  |  |  |  |  |  |  |  |  |  |  |  |  |  |  |  |  |  |  |  |  |  |  |  |  |  |  |  |  |  |  |  |  | 100 |  |  |  |
| *Sucel* | 1 |  |  |  |  |  |  |  |  |  |  |  |  |  |  |  |  |  |  |  |  |  |  |  |  |  |  |  |  | 100 | 100 |  |  |  |  |  |  |  |  |
| *Peta* | - |  |  |  |  |  |  |  |  |  |  |  |  |  |  |  |  |  |  |  |  |  |  |  |  |  |  |  |  |  |  |  |  |  |  |  |  |  |  |
| % in all species | 56 | 7.1 | 1.8 | 1.8 | 8.9 | 1.8 | 16.1 | 1.8 | 23.2 | 10.7 | 1.8 | 1.8 | 1.8 | 1.8 | 1.8 | 7.1 | 1.8 | 1.8 | 1.8 | 16.1 | 3.6 | 1.8 | 1.8 | 1.8 | 1.8 | 1.8 | 1.8 | 1.8 | 1.8 | 1.8 | 1.8 | 1.8 | 1.8 | 3.6 | 1.8 | 1.8 | 3.6 | 1.8 | 1.8 |

| **Table S5. Summary of BLAST search matches for *SLA-DQB1* exon-2 haplotypes identified.** Query coverage and percent identity are indicated for the Sequence ID from the IPD-MHC database and NCBI database. | | | | | | |
| --- | --- | --- | --- | --- | --- | --- |
|  | **IPD-MHC database** | |  | **NBCI** |  |  |
|  | **ID** | **% identity** | **Description** | **ID** | **% identity** | **Description** |
| *DQB1-*1 | [IPDMHCcds:SLA05956](https://www.ebi.ac.uk/ipd/mhc/blast/result/?job=ncbiblast-R20200214-060348-0251-55207636-p2m#result_0) | 65 | *SLA-DQB1**02:05; *SLA-DQB1**02:01 | [KU754587.1](https://www.ncbi.nlm.nih.gov/nucleotide/KU754587.1?report=genbank&log$=nucltop&blast_rank=1&RID=4CB544XM016) | 97.0 | *Sus scrofa* breed Large White and Landrace pig MHC class II antigen (*SLA-DQB1*) mRNA, *SLA-DQB1**0601 allele, complete cds |
| *DQB1-*2 | [IPDMHCcds:SLA05974](https://www.ebi.ac.uk/ipd/mhc/blast/result/?job=ncbiblast-R20200219-011904-0477-42231633-p2m#result_0) | 33.7 | *SLA-DQB1**06:01 | [KU754587.1](https://www.ncbi.nlm.nih.gov/nucleotide/KU754587.1?report=genbank&log$=nucltop&blast_rank=1&RID=4CB544XM016) | 98.2 | *Sus scrofa* breed Large White and Landrace pig MHC class II antigen (*SLA-DQB1*) mRNA, *SLA-DQB1**0601 allele, complete cds |
| *DQB1-*3 | [IPDMHCcds:SLA05981](https://www.ebi.ac.uk/ipd/mhc/blast/result/?job=ncbiblast-R20200214-053025-0682-1552882-p2m#result_0) | 33.9 | *SLA-DQB1**09:01 | [KU754589.1](https://www.ncbi.nlm.nih.gov/nucleotide/KU754589.1?report=genbank&log$=nucltop&blast_rank=1&RID=4C13WT9X01R) | 98.9 | *Sus scrofa* breed Large White pig MHC class II antigen (*SLA-DQB1*) mRNA, *SLA-DQB1**0901 allele, complete cds |
| *DQB1-*4 | [IPDMHCcds:SLA05971](https://www.ebi.ac.uk/ipd/mhc/blast/result/?job=ncbiblast-R20200214-053434-0892-39310595-p2m#result_0) | 34 | *SLA-DQB1**05:02; *SLA-DQB1**05:03 | [KC511016.1](https://www.ncbi.nlm.nih.gov/nucleotide/KC511016.1?report=genbank&log$=nucltop&blast_rank=1&RID=4CB544XM016) | 99.3 | *Sus scrofa* MHC class II antigen (*SLA-DQB1*) mRNA, *SLA-DQB1**0502 allele, complete cds |
| *DQB1-*5 | [IPDMHCcds:SLA05974](https://www.ebi.ac.uk/ipd/mhc/blast/result/?job=ncbiblast-R20200214-055812-0553-57636022-p2m#result_0) | 33.3 | *SLA-DQB1**06:01 | [MF498806.1](https://www.ncbi.nlm.nih.gov/nucleotide/MF498806.1?report=genbank&log$=nucltop&blast_rank=1&RID=4C13WT9X01R) | 98.5 | *Sus scrofa* MHC class II antigen (*DQB1*) mRNA, DQB1*MZ01 allele, complete cds |
| *DQB1-*6 | [IPDMHCcds:SLA09731](https://www.ebi.ac.uk/ipd/mhc/blast/result/?job=ncbiblast-R20200214-054029-0811-1105383-p2m#result_0) | 33.2 | *SLA-DQB1**07:02; *SLA-DQB1**08:04; *SLA-DQB1**08:02; *SLA-DQB1**08:01; SLA; DQB1*07:01:03 | [MF498806.1](https://www.ncbi.nlm.nih.gov/nucleotide/MF498806.1?report=genbank&log$=nucltop&blast_rank=1&RID=4C13WT9X01R) | 96.7 | *Sus scrofa* MHC class II antigen (*DQB1*) mRNA, DQB1*MZ01 allele, complete cds |
| *DQB1-*7 | [IPDMHCcds:SLA08543](https://www.ebi.ac.uk/ipd/mhc/blast/result/?job=ncbiblast-R20200214-054447-0040-17259889-p2m#result_0) | 33 | *SLA-DQB1**08:04 | [KC511019.1](https://www.ncbi.nlm.nih.gov/nucleotide/KC511019.1?report=genbank&log$=nucltop&blast_rank=1&RID=4CB544XM016) | 96.3 | *Sus scrofa* MHC class II antigen (*SLA-DQB1*) mRNA, *SLA-DQB1**BM02 allele, complete cds |
| *DQB1-*8 | [IPDMHCcds:SLA05974](https://www.ebi.ac.uk/ipd/mhc/blast/result/?job=ncbiblast-R20200214-054644-0545-37436448-p2m#result_0) | 33.3 | *SLA-DQB1**06:01 | [KU754587.1](https://www.ncbi.nlm.nih.gov/nucleotide/KU754587.1?report=genbank&log$=nucltop&blast_rank=1&RID=4CB544XM016) | 97.0 | *Sus scrofa* breed Large White and Landrace pig MHC class II antigen (*SLA-DQB1*) mRNA, *SLA-DQB1**0601 allele, complete cds |
| *DQB1-*9 | [IPDMHCcds:SLA09731](https://www.ebi.ac.uk/ipd/mhc/blast/result/?job=ncbiblast-R20200214-054827-0872-17970216-p2m#result_0) | 33.7 | *SLA-DQB1**07:02 | [MF498806.1](https://www.ncbi.nlm.nih.gov/nucleotide/MF498806.1?report=genbank&log$=nucltop&blast_rank=1&RID=4C13WT9X01R) | 98.2 | *Sus scrofa* MHC class II antigen (*DQB1*) mRNA, DQB1*MZ01 allele, complete cds |
| *DQB1-*10 | [IPDMHCcds:SLA05971](https://www.ebi.ac.uk/ipd/mhc/blast/result/?job=ncbiblast-R20200214-054952-0679-98737662-p2m#result_0) | 33.2 | *SLA-DQB1**05:02 | [KC511016.1](https://www.ncbi.nlm.nih.gov/nucleotide/KC511016.1?report=genbank&log$=nucltop&blast_rank=1&RID=4CB544XM016) | 96.7 | *Sus scrofa* MHC class II antigen (*SLA-DQB1*) mRNA, *SLA-DQB1**0502 allele, complete cds |
| *DQB1-*11 | [IPDMHCcds:SLA08543](https://www.ebi.ac.uk/ipd/mhc/blast/result/?job=ncbiblast-R20200214-055106-0333-9661132-p2m#result_0) | 33.2 | *SLA-DQB1**08:04; *SLA-DQB1**08:02; *SLA-DQB1**08:01 | [KC511019.1](https://www.ncbi.nlm.nih.gov/nucleotide/KC511019.1?report=genbank&log$=nucltop&blast_rank=1&RID=4CB544XM016) | 96.7 | *Sus scrofa* MHC class II antigen (*SLA-DQB1*) mRNA, *SLA-DQB1**BM02 allele, complete cds |
| *DQB1-*12 | [IPDMHCcds:SLA09731](https://www.ebi.ac.uk/ipd/mhc/blast/result/?job=ncbiblast-R20200214-055216-0907-6758502-p2m#result_0) | 33.8 | *SLA-DQB1**07:02 | [MF498806.1](https://www.ncbi.nlm.nih.gov/nucleotide/MF498806.1?report=genbank&log$=nucltop&blast_rank=1&RID=4CB544XM016) | 98.5 | *Sus scrofa* MHC class II antigen (*DQB1*) mRNA, DQB1*MZ01 allele, complete cds |
| *DQB1-*13 | [IPDMHCcds:SLA05974](https://www.ebi.ac.uk/ipd/mhc/blast/result/?job=ncbiblast-R20200214-061504-0378-91146748-p2m#result_0) | 33.4 | *SLA-DQB1**06:01 | [KU754587.1](https://www.ncbi.nlm.nih.gov/nucleotide/KU754587.1?report=genbank&log$=nucltop&blast_rank=1&RID=4CB544XM016) | 97.4 | *Sus scrofa* breed Large White and Landrace pig MHC class II antigen (*SLA-DQB1*) mRNA, *SLA-DQB1**0601 allele, complete cds |
| *DQB1-*14 | [IPDMHCcds:SLA08537](https://www.ebi.ac.uk/ipd/mhc/blast/result/?job=ncbiblast-R20200214-064449-0600-62922150-p2m#result_0) | 58.4 | *SLA-DQB1**02:10; *SLA-DQB1**02:05; *SLA-DQB1**02:01 | [KU754584.1](https://www.ncbi.nlm.nih.gov/nucleotide/KU754584.1?report=genbank&log$=nucltop&blast_rank=1&RID=4CB544XM016) | 95.9 | *Sus scrofa* breed Large White and Landrace pig MHC class II antigen (*SLA-DQB1*) mRNA, *SLA-DQB1**0201 allele, complete cds |
| *DQB1-*15 | [IPDMHCcds:SLA05983](https://www.ebi.ac.uk/ipd/mhc/blast/result/?job=ncbiblast-R20200214-055557-0758-45273619-p2m#result_0) | 33.7 | *SLA-DQB1**10:01 | [FJ169380.1](https://www.ncbi.nlm.nih.gov/nucleotide/FJ169380.1?report=genbank&log$=nucltop&blast_rank=1&RID=4CB544XM016) | 98.5 | *Sus scrofa* isolate Sar15 MHC class II antigen (*SLA-DQB*) gene, exon 2 and partial cds |
| *DQB1-*16 | [IPDMHCcds:SLA05983](https://www.ebi.ac.uk/ipd/mhc/blast/result/?job=ncbiblast-R20200214-060511-0788-30814362-p2m#result_0) | 33.2 | *SLA-DQB1**10:01 | [FJ169380.1](https://www.ncbi.nlm.nih.gov/nucleotide/FJ169380.1?report=genbank&log$=nucltop&blast_rank=1&RID=4CB544XM016) | 97.0 | *Sus scrofa* isolate Sar15 MHC class II antigen (*SLA-DQB*) gene, exon 2 and partial cds |
| *DQB1-*17 | [IPDMHCcds:SLA05983](https://www.ebi.ac.uk/ipd/mhc/blast/result/?job=ncbiblast-R20200214-060511-0788-30814362-p2m#result_0) | 33.8 | *SLA-DQB1**10:01 | [FJ169380.1](https://www.ncbi.nlm.nih.gov/nucleotide/FJ169380.1?report=genbank&log$=nucltop&blast_rank=1&RID=4CB544XM016) | 98.9 | *Sus scrofa* isolate Sar15 MHC class II antigen (*SLA-DQB*) gene, exon 2 and partial cds |
| *DQB1-*18 | [IPDMHCcds:SLA05952](https://www.ebi.ac.uk/ipd/mhc/blast/result/?job=ncbiblast-R20200214-060625-0541-59422683-p2m#result_0) | 33.4 | *SLA-DQB1**02:02 | [KU754585.1](https://www.ncbi.nlm.nih.gov/nucleotide/KU754585.1?report=genbank&log$=nucltop&blast_rank=1&RID=4CB544XM016) | 97.4 | *Sus scrofa* breed Large White pig MHC class II antigen (*SLA-DQB1*) mRNA, *SLA-DQB1**0202 allele, complete cds |
| *DQB1-*19 | [IPDMHCcds:SLA09731](https://www.ebi.ac.uk/ipd/mhc/blast/result/?job=ncbiblast-R20200214-054335-0962-61947484-p2m#result_0) | 33.2 | *SLA-DQB1**07:02 | [MF498806.1](https://www.ncbi.nlm.nih.gov/nucleotide/MF498806.1?report=genbank&log$=nucltop&blast_rank=1&RID=4CB544XM016) | 96.7 | *Sus scrofa* MHC class II antigen (*DQB1*) mRNA, DQB1*MZ01 allele, complete cds |
| *DQB1-*20 | [IPDMHCcds:SLA05974](https://www.ebi.ac.uk/ipd/mhc/blast/result/?job=ncbiblast-R20200214-053301-0349-39818665-p2m#result_0) | 33.4 | *SLA-DQB1**06:01 | [KU754587.1](https://www.ncbi.nlm.nih.gov/nucleotide/KU754587.1?report=genbank&log$=nucltop&blast_rank=1&RID=4T1CYY1G014) | 97.4 | *Sus scrofa* breed Large White and Landrace pig MHC class II antigen (*SLA-DQB1*) mRNA, *SLA-DQB1**0601 allele, complete cds |
| *DQB1-*21 | [IPDMHCcds:SLA05974](https://www.ebi.ac.uk/ipd/mhc/blast/result/?job=ncbiblast-R20200214-061654-0717-11005391-p2m#result_0) | 33.2 | *SLA-DQB1**06:01 | [KU754587.1](https://www.ncbi.nlm.nih.gov/nucleotide/KU754587.1?report=genbank&log$=nucltop&blast_rank=1&RID=4CB544XM016) | 96.7 | *Sus scrofa* breed Large White and Landrace pig MHC class II antigen (*SLA-DQB1*) mRNA, *SLA-DQB1**0601 allele, complete cds |
| *DQB1-*22 | [IPDMHCcds:SLA09731](https://www.ebi.ac.uk/ipd/mhc/blast/result/?job=ncbiblast-R20200214-061935-0483-3478434-p2m#result_0) | 33.2 | *SLA-DQB1**07:02 | [MF498806.1](https://www.ncbi.nlm.nih.gov/nucleotide/MF498806.1?report=genbank&log$=nucltop&blast_rank=1&RID=4CB544XM016) | 96.7 | *Sus scrofa* MHC class II antigen (*DQB1*) mRNA, DQB1*MZ01 allele, complete cds |
| *DQB1-*23 | [IPDMHCcds:SLA05971](https://www.ebi.ac.uk/ipd/mhc/blast/result/?job=ncbiblast-R20200214-062201-0026-78910512-p2m#result_0) | 34 | *SLA-DQB1**05:02 | [KC511016.1](https://www.ncbi.nlm.nih.gov/nucleotide/KC511016.1?report=genbank&log$=nucltop&blast_rank=1&RID=4CB544XM016) | 99.3 | *Sus scrofa* MHC class II antigen (*SLA-DQB1*) mRNA, *SLA-DQB1**0502 allele, complete cds |
| *DQB1-*24 | [IPDMHCcds:SLA05971](https://www.ebi.ac.uk/ipd/mhc/blast/result/?job=ncbiblast-R20200214-062325-0123-45877671-p2m#result_0) | 33.8 | *SLA-DQB1**05:02 | [KC511016.1](https://www.ncbi.nlm.nih.gov/nucleotide/KC511016.1?report=genbank&log$=nucltop&blast_rank=1&RID=4CB544XM016) | 98.5 | *Sus scrofa* MHC class II antigen (*SLA-DQB1*) mRNA, *SLA-DQB1**0502 allele, complete cds |
| *DQB1-*25 | [IPDMHCcds:SLA05974](https://www.ebi.ac.uk/ipd/mhc/blast/result/?job=ncbiblast-R20200214-062438-0701-15722427-p2m#result_0) | 33.3 | *SLA-DQB1**06:01 | [KU754587.1](https://www.ncbi.nlm.nih.gov/nucleotide/KU754587.1?report=genbank&log$=nucltop&blast_rank=1&RID=4CB544XM016) | 97.0 | *Sus scrofa* breed Large White and Landrace pig MHC class II antigen (*SLA-DQB1*) mRNA, *SLA-DQB1**0601 allele, complete cds |
| *DQB1-*26 | [IPDMHCcds:SLA05971](https://www.ebi.ac.uk/ipd/mhc/blast/result/?job=ncbiblast-R20200214-062544-0924-64122193-p2m#result_0) | 33.8 | *SLA-DQB1**05:02 | [KC511016.1](https://www.ncbi.nlm.nih.gov/nucleotide/KC511016.1?report=genbank&log$=nucltop&blast_rank=1&RID=4CB544XM016) | 98.5 | *Sus scrofa* MHC class II antigen (*SLA-DQB1*) mRNA, *SLA-DQB1**0502 allele, complete cds |
| *DQB1-*27 | [IPDMHCcds:SLA05974](https://www.ebi.ac.uk/ipd/mhc/blast/result/?job=ncbiblast-R20200214-063037-0429-52502683-p2m#result_0) | 33.3 | *SLA-DQB1**06:01 | [KU754587.1](https://www.ncbi.nlm.nih.gov/nucleotide/KU754587.1?report=genbank&log$=nucltop&blast_rank=1&RID=4CB544XM016) | 97.0 | *Sus scrofa* breed Large White and Landrace pig MHC class II antigen (*SLA-DQB1*) mRNA, *SLA-DQB1**0601 allele, complete cds |
| *DQB1-*28 | [IPDMHCcds:SLA09731](https://www.ebi.ac.uk/ipd/mhc/blast/result/?job=ncbiblast-R20200214-063928-0602-46829010-p2m#result_0) | 33.5 | *SLA-DQB1**07:02 | [MF498806.1](https://www.ncbi.nlm.nih.gov/nucleotide/MF498806.1?report=genbank&log$=nucltop&blast_rank=1&RID=4CB544XM016) | 97.8 | *Sus scrofa* MHC class II antigen (*DQB1*) mRNA, DQB1*MZ01 allele, complete cds |
| *DQB1-*29 | [IPDMHCcds:SLA08542](https://www.ebi.ac.uk/ipd/mhc/blast/result/?job=ncbiblast-R20200214-063328-0248-4710423-p2m#result_0) | 33.7 | *SLA-DQB1**07:01:03; *SLA-DQB1**07:01:02; *SLA-DQB1**07:01:01 | [KU754588.1](https://www.ncbi.nlm.nih.gov/nucleotide/KU754588.1?report=genbank&log$=nucltop&blast_rank=1&RID=4CB544XM016) | 98.2 | *Sus scrofa* breed Landrace pig MHC class II antigen (*SLA-DQB1*) mRNA, *SLA-DQB1**0701 allele, complete cds |
| *DQB1-*30 | [IPDMHCcds:SLA05971](https://www.ebi.ac.uk/ipd/mhc/blast/result/?job=ncbiblast-R20200214-062201-0026-78910512-p2m#result_0) | 34 | *SLA-DQB1**05:02 | [KC511016.1](https://www.ncbi.nlm.nih.gov/nucleotide/KC511016.1?report=genbank&log$=nucltop&blast_rank=1&RID=4CB544XM016) | 99.3 | *Sus scrofa* MHC class II antigen (*SLA-DQB1*) mRNA, *SLA-DQB1**0502 allele, complete cds |
| *DQB1-*31 | [IPDMHCcds:SLA05974](https://www.ebi.ac.uk/ipd/mhc/blast/result/?job=ncbiblast-R20200214-061654-0717-11005391-p2m#result_0) | 33.2 | *SLA-DQB1**06:01; *SLA-DQB1**02:05; *SLA-DQB1**02:01 | [KU754587.1](https://www.ncbi.nlm.nih.gov/nucleotide/KU754587.1?report=genbank&log$=nucltop&blast_rank=1&RID=4CB544XM016) | 96.7 | *Sus scrofa* breed Large White and Landrace pig MHC class II antigen (*SLA-DQB1*) mRNA, *SLA-DQB1**0601 allele, complete cds |
| *DQB1-*32 | [IPDMHCcds:SLA05985](https://www.ebi.ac.uk/ipd/mhc/blast/result/?job=ncbiblast-R20200214-063210-0125-26262688-p2m#result_0) | 93.9 | *SLA-DQB1**11:01 | [L08841.1](https://www.ncbi.nlm.nih.gov/nucleotide/L08841.1?report=genbank&log$=nucltop&blast_rank=1&RID=4CB544XM016) | 98.5 | Swine MHC SLA-DQB allele, b1 domain |
| *DQB1-*33 | [IPDMHCcds:SLA08543](https://www.ebi.ac.uk/ipd/mhc/blast/result/?job=ncbiblast-R20200214-063517-0897-15927994-p2m#result_0) | 33.3 | *SLA-DQB1**08:04; *SLA-DQB1**06:02:02; *SLA-DQB1**08:02; *SLA-DQB1**08:01 | [KC511019.1](https://www.ncbi.nlm.nih.gov/nucleotide/KC511019.1?report=genbank&log$=nucltop&blast_rank=1&RID=4CB544XM016) | 97.0 | *Sus scrofa* MHC class II antigen (*SLA-DQB1*) mRNA, *SLA-DQB1**BM02 allele, complete cds |
| *DQB1-*34 | [IPDMHCcds:SLA08537](https://www.ebi.ac.uk/ipd/mhc/blast/result/?job=ncbiblast-R20200214-064449-0600-62922150-p2m#result_0) | 60.7 | *SLA-DQB1**02:10 | [AB845310.1](https://www.ncbi.nlm.nih.gov/nucleotide/AB845310.1?report=genbank&log$=nucltop&blast_rank=1&RID=4CB544XM016) | 99.6 | *Sus scrofa SLA-DQB1*gene for MHC class II antigen, partial cds, allele: DQB1*02an02 |
| *DQB1-*35 | [IPDMHCcds:SLA05952](https://www.ebi.ac.uk/ipd/mhc/blast/result/?job=ncbiblast-R20200214-063621-0532-6438439-p2m#result_0) | 34.2 | *SLA-DQB1**02:02 | [KU754585.1](https://www.ncbi.nlm.nih.gov/nucleotide/KU754585.1?report=genbank&log$=nucltop&blast_rank=1&RID=4CB544XM016) | 99.6 | *Sus scrofa* breed Large White pig MHC class II antigen (*SLA-DQB1*) mRNA, *SLA-DQB1**0202 allele, complete cds |
| *DQB1-*36 | [IPDMHCcds:SLA08542](https://www.ebi.ac.uk/ipd/mhc/blast/result/?job=ncbiblast-R20200219-060841-0838-74731090-p2m#result_0) | 33.9 | *SLA-DQB1**07:01:03; *SLA-DQB1**07:01:02; *SLA-DQB1**07:01:01 | [KU754588.1](https://www.ncbi.nlm.nih.gov/nucleotide/KU754588.1?report=genbank&log$=nucltop&blast_rank=1&RID=4CB544XM016) | 98.9 | *Sus scrofa* breed Landrace pig MHC class II antigen (*SLA-DQB1*) mRNA, *SLA-DQB1**0701 allele, complete cds |
| *DQB1-*37 | [IPDMHCcds:SLA05956](https://www.ebi.ac.uk/ipd/mhc/blast/result/?job=ncbiblast-R20200214-060223-0327-1245913-p2m#result_0) | 64.2 | *SLA-DQB1**02:05 | [KC511016.1](https://www.ncbi.nlm.nih.gov/nucleotide/KC511016.1?report=genbank&log$=nucltop&blast_rank=1&RID=4CB544XM016) | 95.9 | *Sus scrofa* MHC class II antigen (*SLA-DQB1*) mRNA, *SLA-DQB1**0502 allele, complete cds |
| *DQB1-*38 | [IPDMHCcds:SLA05971](https://www.ebi.ac.uk/ipd/mhc/blast/result/?job=ncbiblast-R20200214-062809-0133-32257546-p2m#result_0) | 32.9 | *SLA-DQB1**05:02 | [KC511016.1](https://www.ncbi.nlm.nih.gov/nucleotide/KC511016.1?report=genbank&log$=nucltop&blast_rank=1&RID=4CB544XM016) | 95.3 | *Sus scrofa* MHC class II antigen (*SLA-DQB1*) mRNA, *SLA-DQB1**0502 allele, complete cds |

| **Table S6. Genetic distance of *SLA-DQB1* exon-2 sequences between bushpig localities and other species.** Genetic distances were calculated using p-distance (base differences per site from averaging over all sequence pairs between groups) in MEGA 7^92^. Only coding positions (1st+2nd+3rd) codons and sites with < 95% coverage were eliminated from analysis. A total of 108 nucleotide sequences and 270 positions in the final dataset were used. | | | | | | | | |
| --- | --- | --- | --- | --- | --- | --- | --- | --- |
| Location | Ankarafantsika | Menabe Antimena | Andasibe-Mantadia | Zimbabwe | KwaZulu-Natal, SA | Soutpansberg, SA | Africa | Other species |
| Ankarafantsika |  | 0.008 | 0.007 | 0.008 | 0.007 | 0.010 | 0.010 | 0.007 |
| Menabe Antimena | 0.037 |  | 0.006 | 0.009 | 0.008 | 0.012 | 0.011 | 0.008 |
| Andasibe-Mantadia | 0.035 | 0.026 |  | 0.009 | 0.008 | 0.011 | 0.012 | 0.009 |
| Zimbabwe | 0.045 | 0.051 | 0.052 |  | 0.008 | 0.009 | 0.010 | 0.007 |
| Soutpansberg, SA | 0.031 | 0.039 | 0.036 | 0.040 |  | 0.009 | 0.010 | 0.007 |
| KwaZulu-Natal, SA | 0.046 | 0.055 | 0.052 | 0.045 | 0.039 |  | 0.013 | 0.009 |
| Africa | 0.052 | 0.052 | 0.058 | 0.050 | 0.049 | 0.063 |  | 0.010 |
| Other species | 0.045 | 0.047 | 0.050 | 0.046 | 0.042 | 0.048 | 0.050 |  |

| **Table S7.** **Summary of *SLA-6* exon-2 haplotypes found in each species and location.** Sampling numbers and haplotype frequencies (%) are based on number of individuals passing quality filtering. Frequencies are provided for each species and location. Suidae and Tayassuidae species are as follows: *Pola*: *P. larvatus, Popo: P. porcus, Phaf: P. africanus, Hyme: H. meinertzhageni, Baba: Babyrousa babyrussa, Susc: S. scrofa, Suba: S. barbatus, Suce:S. celebensis, Peta: P. tajacu.* Shaded haplotypes are those found with 100% BLAST matches on NCBI and the IPD-MHC database (Table S9). Haplotypes whose amino acid translation are identical are shown and labelled ‘AA’ followed by a number. | | | | | | | | |
| --- | --- | --- | --- | --- | --- | --- | --- | --- |
| **Identical amino acid** |  | **AA2** |  |  |  | **AA1** | **AA1** | **AA2** |
| **Location/species** | ***N*** | ***SLA-6* *-*1** | ***SLA-6* *-*2** | ***SLA-6* *-*3** | ***SLA-6* *-*4** | ***SLA-6* *-*5** | ***SLA-6* *-*6** | ***SLA-6* *-*7** |
| **Bushpigs (*Pola*)** | 52 | 98.1 | 1.9 |  |  |  |  |  |
| **Madagascar** | 40 | 100 |  |  |  |  |  |  |
| Ankarafantsika | 28 | 100 |  |  |  |  |  |  |
| Mahajanga Province (general) | 5 | 100 |  |  |  |  |  |  |
| Menabe Antimena | 3 | 100 |  |  |  |  |  |  |
| “East” Madagascar  (Andasibe-Mantadia) | 4 | 100 |  |  |  |  |  |  |
| “North” Madagascar  (Antsiranana Province) | - |  |  |  |  |  |  |  |
| **Mainland** **Africa** | 12 | 91.7 | 8.3 |  |  |  |  |  |
| Zimbabwe | 9 | 100 |  |  |  |  |  |  |
| KwaZulu-Natal, SA | 1 | 100 |  |  |  |  |  |  |
| Soutpansberg, SA | 1 | 100 |  |  |  |  |  |  |
| Tanzania | - |  |  |  |  |  |  |  |
| Africa | 1 |  | 100 |  |  |  |  |  |
| **Other species** |  |  |  |  |  |  |  |  |
| *Popo* | 3 | 66.7 |  |  |  |  |  |  |
| *Phaf* | 1 |  |  |  | 100 |  |  |  |
| *Hyme* | 1 |  |  |  | 100 |  |  |  |
| *Baba* | 1 |  |  |  |  |  |  | 100 |
| *Susc* | 1 |  |  |  | 100 |  |  |  |
| *Suba* | 1 |  |  |  |  |  |  |  |
| *Suce* | 1 |  | 100 |  |  |  |  |  |
| *Peta* | 16 |  |  | 12.5 |  | 18.8 | 81.3 |  |
| **All species** | 73 | 68.3 | 2.6 | 2.6 | 3.9 | 3.9 | 16.9 | 1.3 |

| **Table S8.** **Summary of *BAG6* exon-2 haplotypes found in each species and location.** Sampling numbers and haplotype frequencies are based on number of individuals passing quality filtering. Frequencies are provided for each species and location. Suidae and Tayassuidae species are as follows: *Pola*: *P. larvatus, Popo: P. porcus, Phaf: P. africanus, Hyme: H. meinertzhageni, Baba: Babyrousa babyrussa, Susc: S. scrofa, Suba: S. barbatus, Suce:S. celebensis, Peta: P. tajacu.* Shaded haplotypes are those found with 100% BLAST matches on NCBI (Table S10). haplotypes whose amino acid translation are identical are shown and labelled ‘AA’ followed by a number. | | | | | | | | | |
| --- | --- | --- | --- | --- | --- | --- | --- | --- | --- |
| **Identical amino acid** |  | **AA1** |  | **AA1** |  | **AA1** | **AA1** |  |  |
| **Location/species** | ***N*** | ***BAG6-*1** | ***BAG6-*2** | ***BAG6-*3** | ***BAG6-*4** | ***BAG6-*5** | ***BAG6-*6** | ***BAG6-*7** | ***BAG6-*8** |
| **Bushpigs (*Pola*)** | 51 | 90.2 |  | 3.9 | 2 | 2 |  |  |  |
| **Madagascar** | 39 | 92.3 |  |  |  |  |  |  |  |
| Ankarafantsika | 28 | 92.9 |  |  |  |  |  |  |  |
| Mahajanga Province (general) | 5 | 100 |  |  |  |  |  |  |  |
| Menabe Antimena | 3 | 33.3 |  |  |  |  |  |  |  |
| “East” Madagascar  (Andasibe-Mantadia) | 3 | 100 |  |  |  |  |  |  |  |
| “North” Madagascar  (Antsiranana Province) | - |  |  |  |  |  |  |  |  |
| **Mainland Africa** | 12 | 83.3 | 8.3 | 16.7 | 8.3 | 8.3 |  |  |  |
| Zimbabwe | 9 | 100 |  | 22.2 | 11.1 |  |  |  |  |
| KwaZulu-Natal, SA | 1 |  |  |  |  | 100 |  |  |  |
| Soutpansberg, SA | 1 | 100 |  |  |  |  |  |  |  |
| Tanzania | - |  |  |  |  |  |  |  |  |
| Africa | 1 |  | 100 |  |  |  |  |  |  |
| **Other species** |  |  |  |  |  |  |  |  |  |
| *Popo* | 1 | 66.7 |  |  |  |  |  |  |  |
| *Phaf* | 1 | 100 |  |  |  |  |  |  |  |
| *Hyme* | 1 | 100 |  |  |  |  |  |  |  |
| *Baba* | 1 |  |  |  |  |  | 100 |  |  |
| *Susc* | 1 |  | 100 |  |  |  |  |  |  |
| *Suba* | 1 |  |  |  |  |  |  |  |  |
| *Suce* | 1 |  | 100 |  |  |  |  |  |  |
| *Peta* | 16 |  |  |  |  |  |  | 6.3 | 31.3 |
| **% in all species** | 74 | 65.8 | 2.6 | 2.6 | 1.3 | 1.3 | 1.3 | 1.3 | 6.6 |

| **Table S9. Summary of BLAST search matches for *SLA-6* exon-2 haplotypes identified.** Query coverage and percent identity are indicated for the Sequence ID from the IPD-MHC database. | | | | | | |
| --- | --- | --- | --- | --- | --- | --- |
|  | **IPD-MHC database** |  |  | **NBCI** |  |  |
|  |  | **% identity** | **Description** |  | **% identity** | **Description** |
| *SLA-6-*1 | [IPDMHCcds:SLA09736](https://www.ebi.ac.uk/ipd/mhc/blast/result/?job=ncbiblast-R20200214-015049-0593-98081013-p2m#result_0) | 99.6 | *SLA-6**10:01 | [MH107872.1](https://www.ncbi.nlm.nih.gov/nucleotide/MH107872.1?report=genbank&log$=nucltop&blast_rank=1&RID=4BVUU63N016) | 99.6 | *Sus scrofa* MHC class I antigen (*SLA-6*) mRNA, partial cds |
| *SLA-6-*2 | [IPDMHCcds:SLA06219](https://www.ebi.ac.uk/ipd/mhc/blast/result/?job=ncbiblast-R20200214-011627-0921-93271440-p2m#result_0) | 100 | *SLA-6**08:01; *SLA-6**07:01; *SLA-6**06:01; *SLA-6**05:01; *SLA-6**04:01; *SLA-6**03:01; *SLA-6**02:01; *SLA-6**01:01 | [MF498799.1](https://www.ncbi.nlm.nih.gov/nucleotide/MF498799.1?report=genbank&log$=nucltop&blast_rank=1&RID=4BVUU63N016) | 100 | *Sus scrofa* MHC class I antigen (*SLA-6*) mRNA, *SLA-6**04:01 allele, complete cds |
| *SLA-6-*3 | [IPDMHCcds:SLA09736](https://www.ebi.ac.uk/ipd/mhc/blast/result/?job=ncbiblast-R20200214-011351-0892-575416-p2m#result_0) | 96.3 | *SLA-6**10:01 | [MH107872.1](https://www.ncbi.nlm.nih.gov/nucleotide/MH107872.1?report=genbank&log$=nucltop&blast_rank=1&RID=4BVUU63N016) | 96.3 | *Sus scrofa* MHC class I antigen (*SLA-6*) mRNA, partial cds |
| *SLA-6-*4 | [IPDMHCcds:SLA09736](https://www.ebi.ac.uk/ipd/mhc/blast/result/?job=ncbiblast-R20200214-011516-0987-71705241-p2m#result_0) | 100 | *SLA-6**10:01 | [MH107872.1](https://www.ncbi.nlm.nih.gov/nucleotide/MH107872.1?report=genbank&log$=nucltop&blast_rank=1&RID=4BVUU63N016) | 100 | *Sus scrofa* MHC class I antigen (*SLA-6*) mRNA, partial cds |
| *SLA-6-*5 | [IPDMHCcds:SLA09736](https://www.ebi.ac.uk/ipd/mhc/blast/result/?job=ncbiblast-R20200214-011220-0427-82523302-p2m#result_0) | 96.3 | *SLA-6**10:01 | [MH107872.1](https://www.ncbi.nlm.nih.gov/nucleotide/MH107872.1?report=genbank&log$=nucltop&blast_rank=1&RID=4BVUU63N016) | 96.3 | *Sus scrofa* MHC class I antigen (*SLA-6*) mRNA, partial cds |
| *SLA-6-*6 | [IPDMHCcds:SLA09736](https://www.ebi.ac.uk/ipd/mhc/blast/result/?job=ncbiblast-R20200214-005857-0874-61613116-p2m#result_0) | 96.7 | *SLA-6**10:01 | [MH107872.1](https://www.ncbi.nlm.nih.gov/nucleotide/MH107872.1?report=genbank&log$=nucltop&blast_rank=1&RID=4BVUU63N016) | 96.7 | *Sus scrofa* MHC class I antigen (*SLA-6*) mRNA, partial cds |
| *SLA-6-*7 | [IPDMHCcds:SLA09736](https://www.ebi.ac.uk/ipd/mhc/blast/result/?job=ncbiblast-R20200214-013040-0722-42703687-p2m#result_0) | 98.9 | *SLA-6**10:01 | [MH107872.1](https://www.ncbi.nlm.nih.gov/nucleotide/MH107872.1?report=genbank&log$=nucltop&blast_rank=1&RID=4BVUU63N016) | 98.9 | *Sus scrofa* MHC class I antigen (*SLA-6*) mRNA, partial cds |

| **Table S10. Summary of BLAST search matches for *BAG6* haplotypes identified.** Query coverage and percent identity are indicated for the Sequence ID from the IPD-MHC and NCBI database. | | | |
| --- | --- | --- | --- |
|  | **NBCI** | | |
|  |  | **% identity** | **Description** |
| *BAG6-*1 | [KF941298.1](https://www.ncbi.nlm.nih.gov/nucleotide/KF941298.1?report=genbank&log$=nucltop&blast_rank=1&RID=4BZUKKTX016) | 99.2 | *Sus scrofa* large proline-rich protein *BAG6* transcript variant 1 (*BAG6*) mRNA, complete cds |
| *BAG6-*2 | [KF941298.1](https://www.ncbi.nlm.nih.gov/nucleotide/KF941298.1?report=genbank&log$=nucltop&blast_rank=1&RID=4BZUKKTX016) | 100 | *Sus scrofa* large proline-rich protein *BAG6* transcript variant 1 (*BAG6*) mRNA, complete cds |
| *BAG6-*3 | [KF941298.1](https://www.ncbi.nlm.nih.gov/nucleotide/KF941298.1?report=genbank&log$=nucltop&blast_rank=1&RID=4BZUKKTX016) | 98.93 | *Sus scrofa* large proline-rich protein *BAG6* transcript variant 1 (*BAG6*) mRNA, complete cds |
| *BAG6-*4 | [KF941298.1](https://www.ncbi.nlm.nih.gov/nucleotide/KF941298.1?report=genbank&log$=nucltop&blast_rank=1&RID=4BZUKKTX016) | 98.93 | *Sus scrofa* large proline-rich protein *BAG6* transcript variant 1 (*BAG6*) mRNA, complete cds |
| *BAG6-*5 | [KF941298.1](https://www.ncbi.nlm.nih.gov/nucleotide/KF941298.1?report=genbank&log$=nucltop&blast_rank=1&RID=4BZUKKTX016) | 98.93 | *Sus scrofa* large proline-rich protein *BAG6* transcript variant 1 (*BAG6*) mRNA, complete cds |
| *BAG6-*6 | [KF941298.1](https://www.ncbi.nlm.nih.gov/nucleotide/KF941298.1?report=genbank&log$=nucltop&blast_rank=1&RID=4BZUKKTX016) | 98.93 | *Sus scrofa* large proline-rich protein *BAG6* transcript variant 1 (*BAG6*) mRNA, complete cds |
| *BAG6-*7 | [XM_031688186.1](https://www.ncbi.nlm.nih.gov/nucleotide/XM_031688186.1?report=genbank&log$=nucltop&blast_rank=1&RID=4BZUKKTX016) | 98.08 | PREDICTED: Vicugna pacos BAG cochaperone 6 (*BAG6*), transcript variant X17, mRNA |
| *BAG6-*8 | [XM_007110139.3](https://www.ncbi.nlm.nih.gov/nucleotide/XM_007110139.3?report=genbank&log$=nucltop&blast_rank=1&RID=4BZUKKTX016) | 98.08 | PREDICTED: Physeter catodon BCL2 associated athanogene 6 (*BAG6*), transcript variant X5, mRNA |
